# Interpol review of fingermark composition and visualization 2022-2025

**DOI:** 10.1016/j.fsisyn.2026.100703

**Published:** 2026-06-13

**Authors:** Andy Bécue

**Affiliations:** École des Sciences Criminelles (School of Criminal Justice), Faculté de droit, des sciences criminelles et d’administration publique, Université de Lausanne, Switzerland

## 1 Introduction

A total of 761 papers dedicated to fingermark composition and visualization were collected for this review, covering the period extending between July 2022 and June 2025. This represents almost twice the number of papers collected for those same fields in the previous Interpol report (n = 390) [1]. However, a significant number of those papers suffered from serious shortcomings such as a lack of consideration for forensic needs or specificities, for basic methodological guidelines, or for health and safety issues. Consequently, the decision has been taken to discard 542 of those 761 papers: three in the composition field and 539 in the visualization field (see Section 3.1.1 for further details about this number). The remaining papers (n = 219) were cited and discussed in the different subsections related to fingermark composition (Section 2) and fingermark visualization (Section 3). This decision aims at providing the forensic community with a qualitative critical review, representative of the efforts undergone to improve the discipline.

Also, particular attention has been paid to summarize the numerous studies collected for this report and reflect as accurately as possible the conclusions emitted by the authors. In some cases, a personal note has been added, to emphasize a fact or to make readers aware of a deviation from good practices. Also, a special care has been taken to avoid any transcription error or misunderstanding. Given the high number of papers that were covered, the author of this review asks for leniency if such a mistake is to be found by a cited author.

## 2 Fingermark composition

### 2.1 Overview of the research associated with fingermark composition

For the period covered in this report, 63 papers dedicated to fingermark composition were collected, which represents a +15% increase compared to 2019-2022 (n = 55) [1]. Out of these 63, three papers were discarded for they deviated too much from forensic considerations or could induce health and safety issues – See Section 2.4 for details. To organize this review and emphasize the main research interests and trends, the remaining 60 papers were sorted by their main scope and distributed into the following research fields: “Composition and aging”, “Donor profiling”, “Contaminations”, “Artificial secretions”, and “Miscellaneous”. If the scope of a paper covered different research fields, they were all associated to it. This explains why the numbers provided in the Figures below may deviate from the total number of 60. Readers are encouraged to check the different sections when looking for a technique or a context that could fit in different research fields.

**Fig. 1:**
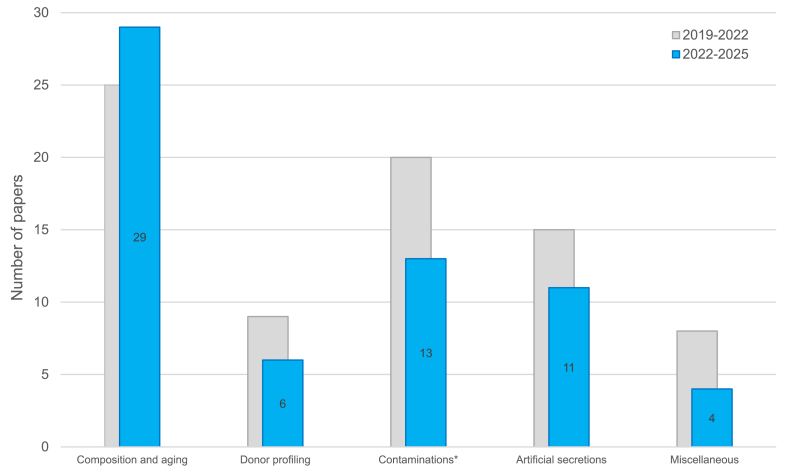
– Histogram representation of the number of papers populating the categories closely linked to fingermark composition. The blue bars and the numbers refer to the 2022-2025 period, whereas the grey bars in the background refer to the 2019-2022 period [1]. The asterisk symbol associated to the Contaminations subcategory means that the reported number is the one remaining after the discarding process (see text for explanations).

**Fig. 2:**
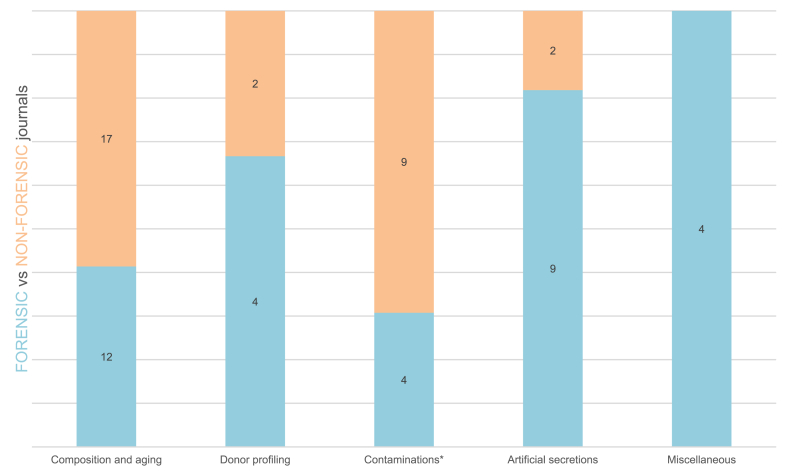
– Histogram representation of the distribution between forensic (blue, bottom) and non-forensic (orange, top) journals, for each of the categories associated with fingermark composition. The labels indicate the number of papers published. The asterisk symbol associated to the Contaminations subcategory means that the reported number is the one remaining after the discarding process (see text for explanations).

To get an overview of the 2022-2025 research trends, the numbers of papers populating the research fields described above have been plotted and compared to 2019-2022 [1] – see Fig. 1. At the exception of “Fingermark composition and aging” (+16%), all the research fields related to fingermark composition experienced a decline in the number of publications. This trend is further discussed in Section 3.1.1, for it is more pronounced for the fingermark visualization field. In terms of research interests, gathering information about the composition of fingermarks and aging processes remains the most popular topic (29 papers), followed by contaminations (13 papers) and artificial secretions (11 papers). In terms of journal scopes, a perfect split was observed between forensic journals (31 papers) and non-forensic ones (29 papers) – see Fig. 2. Both trends were observed for the 2019-2022 period [1]. The success of non-forensic journals can be explained by the technological links associated with composition analysis, which could result in analytical methods prevailing over forensic considerations in some studies.

A recurrent limitation of the studies performed on fingermark composition are the very limited sample sets (e.g., one donor, few depositions, sebum-rich secretions, excessive spiking). Most of the studies reviewed in the forthcoming sections must hence be considered as preliminary/pilot, and the observed trends or the expressed conclusions taken with caution. Few papers considered natural fingermarks left on realistic substrates or exposed to casework-like conditions. This is certainly an aspect of the field that could be improved to strengthen the conclusions and promote the transposition to the operational field. In this context, the IFRG guidelines [2] were cited by three papers, only. Although these guidelines are primarily intended for people evolving in the visualization field, they would assuredly benefit equally the people studying the composition of fingermarks (e.g., donors, types of secretions, substrates).

### 2.2 Composition and aging

**Foreword** – Firstly, most of the papers related to the determination of fingermark composition or the estimation of the time since deposition were performed on ideal sets of fingermarks (e.g., enriched and/or extremely fresh fingermarks, uncommon or pristine substrates, unrealistic collection protocols), far from actual forensic traces or casework conditions. Those studies should hence be taken as proof-of-concepts, and the emitted conclusions taken with extreme caution. Secondly, some of the papers cited below are associated with the medical field or lipidomic research. They have somewhat been included in this report because they involved fingermark deposition and analysis, and may hence be of interest for the forensic community.

**Composition in amino acids** – Composition in amino acids of eccrine-rich fingermarks left on plastic sheets, using UHPLC-MS/MS, to eventually characterize or differentiate donors [3]. The authors emphasized the impact of the amino acids contained in some porous substrates (e.g., white paper, newspaper, Kraft paper) on the resulting composition. For this reason, they recommended using non-porous substrates (i.e., plastic sheet) to perform this kind of study in laboratory conditions.

**Composition in lipids** – Determination of the impact of a firing event on the lipid composition of fingermarks left on cartridge cases; application to lipid spots and sebum-rich fingermarks left on cartridge cases that were analysed with GC-MS/MS [4]. Use of one-dimensional and two-dimensional GC-MS approach to analyse sebum-rich fingermarks left on glass slides [5]. Composition in triacylglycerols and wax esters of sebum-rich fingermarks left on aluminium foil, using ESI-MS [6,7]. These authors emphasized the impact of the grooming site on the fingermark composition, with differences observed between the cheek and forehead, compared to the neck. Comparison of derivatization methods for the analysis of sebum-rich fingermarks left on microfiber filter and glass slides with GC-MS [8]. Assessment of sample storage conditions for sebum-rich fingermarks left on aluminium foil and analysed by LC-MS, in the context of lipidomic research [9]. Comparison of the compositions in lipids of natural and sebum-rich fingermarks, considering one-day-old fingermarks left on office paper and analysed with GC-MS [10]. On both types of secretions, the most abundant lipids were squalene, palmitic acid and cholesterol. The authors also emphasized a correlation between six compounds (i.e., cholesterol, myristic acid, palmitoleic acid, stearyl palmitoleate, squalene and pentadecanoic acid). Finally, sebum-rich fingermarks were characterized by a higher content in lipids (i.e., four times on average), with varying proportions according to their nature. On the contrary, natural fingermarks were characterized by less lipids and more variable compositions.

**Overall composition** – Composition analysis of fresh natural fingermarks collected over a one-year timespan, using MALDI-MS, to gain knowledge about donor intravariability [11] and intervariability [12]. Use of silver NP in suspension to analyse the composition of fingermarks using MALDI-MS and SALDI-MS [13]. Presentation of the use of metal-assisted LDI MS imaging to probe the molecular composition of fingermarks [14].

**Aging processes** – Monitoring of lipid degradation through the combined use of MALDI-MS and Kendrick mass defect plot analysis; application to sebum-rich fingermarks left on glass slides by a single donor [15]. Monitoring of the cholesterol/squalene evolution over time by GC-MS; application to sebum-rich fingermarks left on polystyrene and processed with Fe-BPS [16]. Colour contrast modifications over time to determine whether the sex of the donor influences the fingermark aging processes; application to sebum-rich fingermarks left on glass or polystyrene and dusted with TiO_2_ powder [17]. Monitoring of microbiome modification (e.g., diversity and abundance) over time using DNA sequencing; application to natural fingermarks left on glass slides [18]. Monitoring of ridge modifications (i.e., height and clarity) over time using an optical profilometer and TiO_2_ powder; application to sebum-rich fingermarks left on plastic [19] and glass slides [[19], [20], [21]]. These authors recognized their studies were at the proof-of-concept stage. However, it can be noted that greater ridge height losses were observed for fingermarks left on plastic and exposed to natural light (as opposed to glass and darkness, respectively).

**Time since deposition** – Determination of the age of a fingermark by monitoring the decay of unsaturated triacylglycerols with MALDI-MS; application to fingermarks left on glass [22]. The authors concluded that the significant person-to-person variations in unsaturated lipids constitutes a major limitation of this approach. Indeed, the model needs to be corrected for each individual to increase the prediction accuracy. Determination of the age of a fingermark through the combined use of NIR-HSI and individual aging patterns; application to sebum-rich fingermarks left on glass slides [23]. [Note: it is understood from the paper that the authors considered that the “preservation” of the fingermarks lifted with a tape and deposited on acetate sheet applied to the composition rather than to the ridge pattern.] Determination of the age of bloody fingermarks through transfer on PVDF membranes combined with SECM analysis of the alkaline phosphatase activity [24,25]. Determination of the age of sebum-rich fingermarks through transfer on nitrocellulose membranes combined with SECM analysis of the lipid oxide degradation [26]. Determination of the age of fingermarks using tape-lifting and DESI-MS imaging; application to (natural) fingermarks left on glass and aged for up to 15 days before being dusted with BMP [27].

**Reviews** – Extensive review about the analysis of organic constituents found in fingermarks [28]. The authors identified a total of 19 studies, selected for the clarity of their methodology. Those studies reported the detection of lipids (squalene being the most common one) and/or amino acids (alanine, glycine, leucine, lysine and serine being the most common ones) in fingermarks deposited on various substrates (e.g., glass, plastic, aluminium or paper) and analysed mostly with GC-MS. Review about the analysis of sebum from the skin and fingermarks using ESI-MS [29]. Review about the application of lipidomic in forensic science, including the fingermark field [30]. Review about the role of MALDI-MS and SALDI-MS in the chemical analysis of fingermarks [31].

Acronyms used: **BMP** (black magnetic powder), **DESI** (desorption electrospray ionisation), **DNA** (deoxyribonucleic acid), **ESI** (electrospray ionisation), **Fe-BPS** (iron oxide-based black powder suspension), **GC** (gas chromatography), **HSI** (hyperspectral imaging), **LC** (liquid chromatography), **LDI** (laser desorption ionisation), **MALDI** (matrix-assisted laser desorption ionisation), **MS** (mass spectrometry), **NIR** (near infrared), **NP** (nanoparticle), **PVDF** (poly-vinylidene fluoride) **SALDI** (surface-assisted laser desorption ionisation), **SECM** (scanning electrochemical microscopy), **UHPLC** (ultra-high performance liquid chromatography).

### 2.3 Donor profiling

**Foreword** – Most of the papers related to donor profiling were based on ideal sets of fingermarks (e.g., enriched and fresh fingermarks, unrealistic collection conditions), which are likely to result in an overestimation of the performance of the reported approaches. For these reasons, most of the cited studies are still deemed as being at a preliminary/pilot stage and were not further detailed.

**Age** – Determination of the age of donors from the content in fatty acids of their fingermarks; use of fresh and sebum-rich fingermarks left on glass and analysed with GC-MS [32]. The authors showed that younger donors (i.e., <20-year-old) were characterized by short-chained saturated fatty acids (i.e., ten carbons or less), whereas older donors (i.e., >60-year-old) presented long-chained ones (i.e., twenty carbons or more). It was also shown that the content in pentadecanoic, oleic, and arachidic acids increases with the age before starting to decrease at middle age.

**Geographical origin** – Determination of the geographical origin of individuals from the relative content of amino acids, considering eccrine-rich fingermarks left on plastic sheets and analysed with UHPLC-MS/MS [33].

**Sex** – Determination of the sex of individuals from the content in C_18_ and C_20_ alcohols and in branched fatty acids; use of sebum-rich fingermarks collected on glass slides and analysed with GC-MS [34]. Same objective considering eccrine-rich fingermarks collected on plastic sheets and analysed with UHPLC-MS/MS [3]. In the same context, the composition of sebum-rich fingermarks in triacylglycerols and wax esters does not appear to be substantially impacted by the sex of the donor [7].

**Smoking habits** – Differentiation between smokers and non-smokers by considering sebum-rich fingermarks left by male individuals on aluminium and analysed with ATR-FTIR [35].

Acronyms used: **ATR** (attenuated total reflection), **FTIR** (Fourier transform infrared), **GC** (gas chromatography), **MS** (mass spectrometry), **UHPLC** (ultra-high performance liquid chromatography).

### 2.4 Contaminations

**Foreword** – Most of the papers related to contaminated fingermarks were based on limited sets of fingermarks (e.g., one donor, sebum-rich fingermarks, unrealistic contamination scenarios), which are likely to result in an overestimation of the performance of the reported approaches. For these reasons, most of the cited studies are still deemed as being at a preliminary/pilot stage and were not further detailed. Additionally, three papers were not cited here-below for they deviate too much from forensic considerations or could induce health and safety issues.

**Cosmetics** – Analysis of cosmetic-contaminated fingermarks left on non-porous substrates, using magnetic NPs in solution combined with SALDI-MS [36].

**Drugs** – Impact of time and temperature on drug-contaminated fingermarks, using Raman spectroscopy [37]. Impact of a surface contamination with amphetamine before and after fingermark deposition, using powder dusting [38] combined with FESEM [39]. Computational simulation of the use of IND and DFO as colorimetric tests to detect amphetamine in contaminated fingermarks [40]. Chemical imaging of methamphetamine-spiked fingermarks left on a silicon substrate, using SERS [41]. Detection of drugs and drug metabolites in drug consumers' fingermarks left on glass slides and analysed by LC-MS/MS [42]. Detection of drugs in contaminated fingermarks left on glass slides and analysed with thermal desorption DBDI-MS [43] or Raman spectroscopy [44]. Analysis of drug-contaminated fingermarks by swabbing drug consumers’ smartphones followed by UHPLC-MS/MS [45]. Imaging of antipsychotics-contaminated fingermarks using MALDI-MRT-MS [46] and a combination of MALDI-MS, DESI-MS, and SICRIT [47]. In this last study, the compatibility between the fingermark detection techniques (i.e., IND/Zn, DMAC) and the MALDI or DESI MS imaging processes, applied subsequently, has been confirmed.

**Explosives** – van Damme et al. conducted a prevalence study to establish the natural occurrence of 16 inorganic ions related to explosives on the hands of people [48]. To reach that goal, the authors swabbed the hands of 297 participants (201 in Europe and 96 in the USA) and analysed those with IC-MS. The results showed that some ions are commonly present in varying quantities (i.e., chloride, potassium, sodium, sulphate, nitrate, magnesium, calcium, phosphate, ammonium), mostly due to their presence in natural sweat or in household products. The authors also observed that perchlorate ions can also be detected in low quantities despite the absence of deliberate handling of such chemicals. On the other hand, thiocyanate, chlorate, nitrite, lithium, strontium, and barium were rarely detected, and if so, in low quantities.

Acronyms used: **DBDI** (dielectric barrier discharge ionisation), **DESI** (desorption electrospray ionisation), **DFO** (1,8-diazafluoren-9-one), **DMAC** (4-dimethylaminocinnamaldehyde), **FESEM** (field emission scanning electron microscopy), **IC** (ion chromatography), **IND** (1,2-indanedione), **IND/Zn** (1,2-indanedione combined with zinc chloride), **LC** (liquid chromatography), **MALDI** (matrix-assisted laser desorption ionisation), **MRT** (multi reflecting time-of-flight), **MS** (mass spectrometry), **NP** (nanoparticle), **SALDI** (surface-assisted laser desorption ionisation), **SERS** (surface-enhanced Raman spectroscopy), **SICRIT** (soft ionisation by chemical reaction in transfer), **UHPLC** (ultra-high performance liquid chromatography).

### 2.5 Artificial secretions

**Foreword** – The use of artificial secretions in the fingermark detection field can be debatable, but they proved their usefulness for quality control (e.g., test strips and proficiency tests) or teaching purposes. If the use of commercially available pads has been proved to be unreliable, as described in the previous report [1], most of the studies below described the use of home-made artificial secretions designed to meet their respective objectives.

**Amino acids in solution** – Dilutions series of a solution containing 14 amino acids, combined with a Fujifilm Dimatix Materials Printer, were used to assess the decrease of luminescence of IND/Zn-processed items stored in different conditions [49]. Dilutions series of amino acid solutions were also used to generate spot tests aiming at assessing the shelf life of IND/Zn working solution [50] or to impregnate filter paper used to assess the impact of carrier solvents on the reactivity of IND/Zn and NIN [51]. A solution of L-alanine mixed with semen was used in combination with a stamp to generate fingermarks in a reproducible manner [52].

**Artificial sebum** – Rosik et al. reviewed the artificial sebum formulations that can be found in various research areas, including forensic science [53]. The composition and the physicochemical properties of those artificial secretions were thoroughly discussed and compared to human sebum. With more than 80 formulations identified, this paper constitutes a precious source of information for people willing to use artificial sebum in their research projects.

**Artificial emulsion** – Steiner et al. presented the second part of their study aiming at producing realistic artificial fingermarks [54]. The authors combined artificial eccrine sweat and sebum to create an emulsion, which was further used to fill a modified inkjet printer cartridge. Inked fingerprints were then used as models for the printer to generate artificial fingermarks on office paper and transparent acetate sheets. After 24 h of aging, the printed fingermarks were processed with conventional detection techniques (i.e., IND/Zn, NIN, ORO, PD, CA fuming – R6G, VMD_Au/Zn_, and powder dusting), applied alone or in sequence. On both substrates, the artificial fingermarks behaved quite similarly to actual fingermarks when the techniques were applied alone, at the exception of PD (no detection) and VMD (empty marks, mostly). The application in sequence resulted in slight variations compared to actual fingermarks, with a more pronounced contrast and quality loss. Also, the fingermarks printed on acetate sheets were characterized by a dotty aspect of their ridges, caused by the printing process and differentiating them visually from actual fingermarks. Finally, a preliminary immersion study showed that artificial fingermarks behaved similarly to actual ones, with amino acid reagents unable to detect the immersed marks as opposed to ORO.

**Artificial contact traces** – Arsenault et al. proposed to combine the use of an artificial emulsion with dilution series of (trout and mouse) DNA to investigate DNA transfer, persistence and recovery [55]. Artificial fingermarks were generated by spreading the artificial mixture over a fingertip before touching a glass slide. If not directly related to fingermark detection, this study was deemed relevant to appear in this report.

**Other** – Commercially available artificial secretions (i.e., eccrine sweat, sebum, and emulsion) were used to better understand the impact of finger secretions on touchscreens [56]. If not directly linked to fingermark detection, this paper allows getting a glimpse at the interaction mechanisms between a fingertip and a touched surface.

**Developed in other sections** – Use of a commercial pad containing sebaceous oils, amino acids and salts to generate fingermarks and investigate the impact of a thermal layer removal process [57] or a detonating IED [58] – see Sections 3.4.5, 3.5.4, respectively. Use of amino acids in solution and artificial emulsion to create positive control tests for fingermark detection techniques [59] – see Section 3.6.1.

Acronyms used: **CA** (cyanoacrylate), **IED** (improvised explosive device), **IND/Zn** (1,2-indanedione combined with zinc chloride), **NIN** (ninhydrin), **ORO** (Oil red O), **PD** (physical developer), **R6G** (Rhodamine 6G), **VMD** (vacuum metal deposition).

### 2.6 Miscellaneous

The performance of an optical portable device (i.e., Ridgeway, from Intelligent Fingerprint Ltd – UK) able to quantify the amount of secretions left on a glass slide was investigated by Pollard & Wolf [60]. On that aspect, the authors confirmed that a strong correlation exists between the mass of a fingermark and the score provided by the device. Initially developed for drug testing, the device was used in this study to investigate different parameters linked to the deposition mechanism (e.g., intra- and inter-variability, hand washing, use of gloves, cumulative deposition, etc.).

The impact of surface properties (i.e., roughness, topography, wettability, and free energy) on the transfer of (natural) secretions from a fingertip to a non-porous substrate (i.e., PP and aluminium) was investigated by Hughes et al. [61]. The authors observed that the deposit conforms to the shape of the substrate, regardless of the surface roughness. As a result, the surface roughness of the touch deposit increases with the surface roughness. They also observed that the height profile of the surface increases consistently after the fingermark deposition, regardless of the surface roughness, with an average of +0.26 μm and +0.36 μm for PP and aluminium, respectively. In terms of wettability, aluminium was characterized by a greater wettability compared to PP, meaning that those substrates would present a greater adhesion affinity for hydrophilic secretions (e.g., eccrine-rich fingermarks) and lipophilic secretions (e.g., sebum-rich fingermarks), respectively.

The impact of depletion series on the quality of fingermarks was investigated by De Alcaraz-Fossoul et al., considering sebum-rich fingermarks left on glass slides and dry-dusted using BMP or TiO_2_ powder, before being characterized using ridge clarity metrics [21,62] and height variations [21].

Acronyms used: **BMP** (black magnetic powder), **PP** (polypropylene).

## 3 Fingermark visualization

### 3.1 Overview of the research associated with fingermark visualization

#### 3.1.1 Research trends and interests

For the period covered in this report, 714 papers dedicated to fingermark visualization were collected, which represents a substantial increase of +68% compared to 2019-2022 (n = 425) [1]. To help organizing this report and emphasizing the research interests and trends, those papers were sorted by their scope(s) and distributed into the following research fields: “Observation and recording methods” (I/), “Studies focused on detection techniques” (T/), “Studies focused on substrates” (S/), “Studies focused on contextual situations” (C/), “Quality assessment” (Q/), and “Impact of detection techniques on other forensic traces” (X/). Subcategories were also created to further dispatch the papers and organize the reviewing process (e.g., “T/Amino acid reagents”, “T/Cyanoacrylate fuming”, …, “S/Metal and cartridges”, “S/Adhesives and tapes”, …). Where possible, the papers were associated with only one research field, characterizing the main objective of the study. But in some cases, the objectives were multiple and clearly identified, resulting in a same paper distributed into more than one research field. For example, a study aiming at determining the best way to detect fingermarks on banknotes would have been associated to “S/Banknotes”, but not in any of the subcategories related to the detection techniques reported in the paper. On the contrary, the specific application of Oil Red O on papers immersed in water would have been associated to “T/Lipid stains” and “C/Exposition to liquids”. This explains why the numbers provided in the text and Figures below may deviate from the total number of 714. The readers are encouraged to check the different sections when looking for a reagent or a context that could fit in different research fields.

**Fig. 3:**
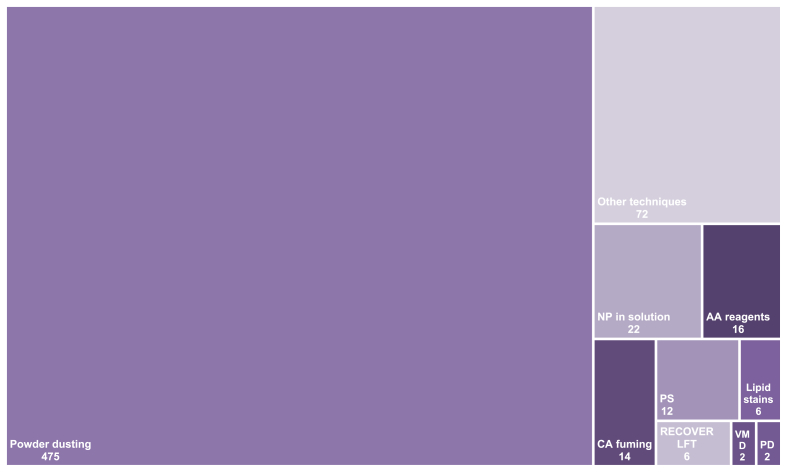
– Treemap representation of the number of papers primarily dedicated to fingermark detection techniques, when considering all the papers populating the category “T/Studies focuses on detection techniques”. The area of each box is directly related to the number of papers associated with each technique, which is also reported below the labels. Acronyms used: AA (amino acid), CA (cyanoacrylate), NP (nanoparticle), PD (physical developer), PS (powder suspension), VMD (vacuum metal deposition).

To get a first glimpse of the 2022-2025 research trends, the numbers of papers populating the category “T/Studies focused on detection techniques” have been plotted in a treemap and compared to 2019-2022 [1] – see Fig. 3. As it can be seen, “Powder dusting” clearly outclasses the other detection techniques, with 475 papers on its own. This represents 67% of the papers dedicated to fingermark visualization (n = 714) and a substantial increase compared to the 2019-2022 period (n = 188; 44% of the 425 papers dedicated to fingermark visualization). The apparent overwhelming success for powder dusting can be explained by the fact that it is the easiest detection technique to carry out in a research environment, as it requires a brush and fingermarks deposited on a non-porous substrate. Unfortunately, for most of those papers, fingermark detection capability was clearly taken as a pretext for the synthesis and characterization of optically active materials – without any consideration for forensic needs and specificities, basic methodological guidelines, or health and safety issues. We hence decided to discard 446 of those papers and only kept 29 for “T/Powder dusting” – see Section 3.3.6 for further details. The same policy applied to “T/Powder suspension” (12 papers collected, 3 discarded), “T/Nanoparticles in solution” (22 papers collected, 15 discarded), “T/Other techniques” (72 papers collected, 64 discarded), “I/Advanced imaging” (14 papers collected, 3 discarded). Out of the 17 collected reviews covering fingermark visualization, only five were cited. Overall, 539 papers were discarded, out of the 714 that were collected for the 2022-2025 period. As a result, 175 papers remained, and their content taken into consideration for this critical review. This decision was motivated by the significant increase of papers published in the field during the covered period and the need to provide the forensic community with a qualitative critical review accurately representing the efforts undergone to improve the discipline.

**Fig. 4:**
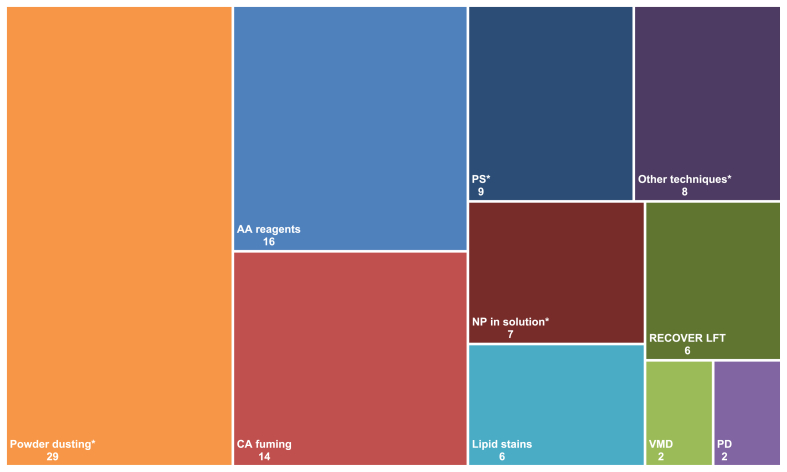
– Treemap representation of the number of papers primarily dedicated to fingermark detection techniques and kept for the report (i.e., after the removal of the discarded papers for those accompanied with an asterisk symbol). The area of each box is directly related to the number of papers associated with each technique (and reported on the chart). Acronyms used: AA (amino acid), CA (cyanoacrylate), NP (nanoparticle), PD (physical developer), PS (powder suspension), VMD (vacuum metal deposition).

When updating the treemap chart, a more accurate view of the shared interests between the different detection techniques does emerge (see Fig. 4). Powder dusting remains the most popular detection technique (29 papers), followed by amino acid reagents (16 papers), cyanoacrylate fuming (14 papers) and powder suspension (9 papers).

**Fig. 5:**
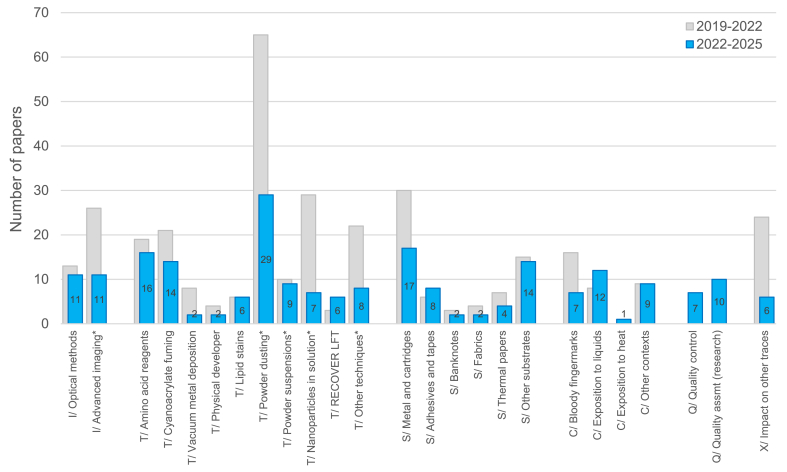
– Histogram representation of the number of papers populating each of the subcategories associated with fingermark visualization. The blue bars and the numbers refer to the 2022-2025 period, whereas the grey bars in background refer to the 2019-2022 period [1]. The asterisk symbol associated to some subcategories means that the reported numbers are those remaining after the discarding process (see text for explanations).

To get an overview of the 2022-2025 research trends, the numbers of papers populating the research fields described above have been plotted and compared to 2019-2022 [1] – see Fig. 5. The striking observation that can be made is that, despite the substantial increase of papers published during the 2022-2025 period (i.e., 714 – compared to 425 for the 2019-2022 period), almost all the research fields dedicated to fingermark visualization experienced a decline in the number of publications. The only exceptions are “T/Lipid stains” (equal number), “T/RECOVER LFT” (+100%), “S/Adhesives and tapes” (+33%), and “C/Exposition to liquids” (+50%). If we exclude “T/Powder dusting”, “T/Nanoparticles in solution”, and “T/Other techniques”, the most significant drops were experienced by “T/Vacuum metal deposition” (−75%), “I/Advanced imaging” (−58%), “C/Bloody fingermarks” (−56%), and “S/Metal and cartridges” (−43%). Such a decline appears counter-intuitive with regards to the substantial increase of papers published during the 2022-2025 period. One of the explanations relies in the fact that most of the additional papers were dedicated to powder dusting, as explained above. Another explanation could be linked to the COVID crisis, with several research projects forced to slow down (or stop) during the 2019-2022 period, delaying the gathering and publication of results. Finally, this decline could also be the consequence of a deterioration of the worldwide research environments (e.g., decrease in funding possibilities, change in research policies, work overload).

#### 3.1.2 Publication strategies

**Fig. 6:**
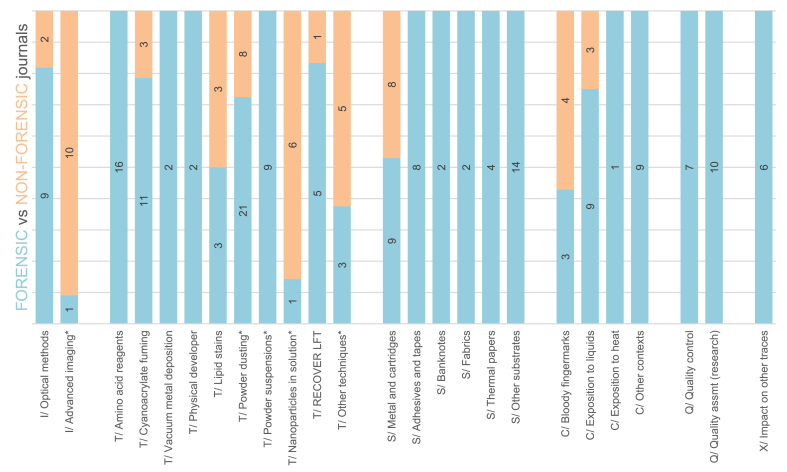
– Histogram representation of the distribution between forensic (blue, bottom) and non-forensic (orange, top) journals, for each of the subcategories dedicated to fingermark visualization and considered in this report. The labels indicate the number of papers published. The asterisk symbol associated to some subcategories means that the reported numbers are those remaining after the discarding process (see text for explanations).

Overall, out of the 714 papers collected, 140 were published in forensic journals (20%) and 574 in non-forensic journals (80%). In the last report, the ratio was of 161 papers (38%) and 264 papers (62%), respectively. The figures obtained in this report are mostly explained by the substantial increase of publications related to powder dusting in non-forensic journals, rather than a loss of interest for forensic journals. Indeed, when focusing on the papers retained for this critical review, most of the studies covering the different aspects of fingermark visualization were published in forensic journals, ensuring to target the forensic readership (see Fig. 6). Two exceptions stand out: “I/Advanced imaging” and “T/Nanoparticles in solution”, which were mostly published in non-forensic journals (mostly chemistry-oriented ones). This can be explained by the technological aspects of those research topics, with analytical methods sometimes prevailing over forensic considerations.

**Fig. 7:**
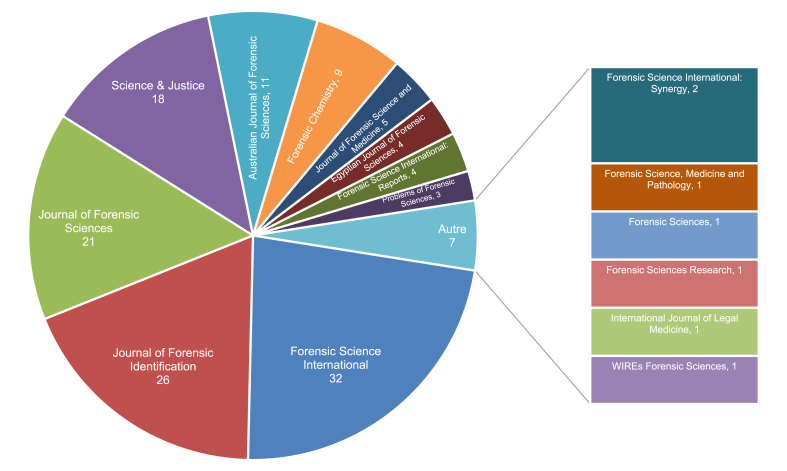
– Pie chart representation of the number of papers dealing with fingermark visualization and published in forensic journals.

*Forensic Science International* (32 papers), *Journal of Forensic Identification* (26 papers), and *Journal of Forensic Sciences* (21 papers) represent 56% of the papers published in forensic journals – see Fig. 7. The same journals already constituted the Top 3 for the 2019-2022 period, with 37, 37, and 20 papers, respectively [1]. With regards to non-forensic journals, the most popular ones were also those presenting the highest numbers of discarded papers (see Table 1). After the discarding process, *Analyst* (3 papers), *Microchemical Journal* (3 papers), and *Scientific Reports* (3 papers) represented the three most popular non-forensic journals cited in the context of fingermark visualization.

**Table 1:** – List of the ten most popular non-forensic journals dealing with fingermark visualization, accompanied by the number of published papers, of discarded ones, and of those included in this report (see text for explanations).

Journal names	# of published papers	# of discarded papers	# of remaining papers
Journal of Luminescence	26	26	-
Inorganic Chemistry Communications	23	23	-
Journal of Alloys and Compounds	23	23	-
Ceramics International	19	19	-
Journal of Molecular Structure	18	18	-
Journal of Photochemistry and Photobiology A	17	16	1
Spectrochimica Acta Part A	17	17	-
Materials Research Bulletin	16	16	-
ChemistrySelect	15	14	1
Dyes and Pigments	15	15	-

Given the extent of the publication rate related to powder dusting (n = 475), we decided to investigate further the publication strategies linked to the discarded papers (n = 446). First, by differentiating the papers according to the powder particle size, it appears that the dusting of nanoparticles (305 discarded papers) outclasses microparticles (141 discarded papers). Such practice goes against all ethical considerations for practitioners in terms of health and safety issues, as well as against any scientific strategy dedicated to fingermark detection. Unfortunately, this trend had already been reported in the last two Interpol reports [1,63] and does not seem to weaken. Unsurprisingly, most of the papers that were discarded were published in non-forensic journals (i.e., 98%). The only exceptions are papers published in the Egyptian Journal of Forensic Sciences (3 papers), Journal of Forensic Sciences (2 papers), Forensic Chemistry (1 paper), and Science and Justice (1 paper). These seven papers involved or promoted the dusting of nanoparticles, sometimes after having voluntarily milled micron-sized powders up to the nanoscale. For microparticles, the 141 discarded papers were published in 66 different journals, representing an average of 2.1 papers per journal. For nanoparticles, the 305 discarded papers were published in 117 different journals, for an average of 2.6 papers per journal. This reflects the fact that the authors of those papers follow no specific publication strategy other than an opportunistic one combined with publications in predatory journals.

#### 3.1.3 Good practices

A detailed methodology of the way the fingermarks were collected and processed is crucial in a paper dealing with fingermark visualization, as it is known that the substrates, donors, and aging conditions may strongly impact the performance of a detection technique. Several papers describe good practices to adopt in this research field, among which the IFRG guidelines [2], openly available to researchers (https://www.theiai.org/docs/JFI-2014-02-174.pdf). For the 2022-2025 period, the IFRG guidelines were cited by 83 papers dealing with fingermark visualization, representing 12% of the total number of papers collected (or 34% after the discarding process). In absolute numbers, this trend is stable compared to the 2019-2022 period (n = 90).

If the IFRG guidelines were correctly cited in most papers, minor deviations were observed: 13 papers cited erroneous page numbers (i.e., “174-197” instead of “174-200”) and three papers omitted the author field. A more striking observation is the fact that the IFRG guidelines were cited as a kind of *alibi* by 13 papers (e.g., “as per the guidelines of the IFRG”) without providing any reference, methodological details, nor explanation about the signification of the acronym. The IFRG guidelines were cited in relationship with the fingermark collection (52 papers), the research phase number (35 papers), the grading scales (31 papers), or the technological readiness level (1 paper). It should be noted that the IFRG guidelines could have been cited multiple times in a paper, explaining why the sum of those numbers exceeds 83.

Regarding fingermark collection: out of the 52 papers that cited the IFRG guidelines for this purpose, 45 (87%) referred to the use of natural fingermarks (or secretions). This means that most of the authors were aware of the specificities associated with this kind of fingermarks. However, if 32 papers proposed a correct methodology to produce natural fingermarks, some deviations were witnessed for 13 papers, which represents almost one third of the papers referring to the use of natural fingermarks. The deviations were the following: no information about how the so-called “natural” fingermarks were produced (8 papers), fingermark deposition 2 – 5 min after having washed the hands (3 papers), indication that the donors were prevented to touch anything for 20 min after having washed their hands (1 paper), and erroneous protocol as the donors were requested to purposedly touch their nose and forehead before depositing fingermarks (1 paper).

Regarding the research phase: the IFRG guidelines were cited in the frame of Phase 1 – Pilot studies (19 papers), Phase 2 – Optimization and comparison (13 papers), and Phase 3 – Validation via pseudo-operational trials (5 papers). It should be noted that two papers encompassed multiple phases, explaining why the sum of those numbers exceeds 35.

Overall, it can be concluded that the use of natural fingermarks and the awareness about the IFRG guidelines contribute to strengthen the research in the fingermark visualization field.

### 3.2 Observation and recording methods

#### 3.2.1 Optical methods

**Preliminary/Pilot studies** – Use of a 92-LED lighting dome to capture fingermarks on curved items while automatically correcting the distortions induced by the surface curvature [64]. Combined use of hyperspectral data and component analysis to separate overlapped fluorescent fingermarks [65]. Combination of an industrial camera with high-power LEDs to record fingermarks (and other forensic traces) at long distance [66]. Use of Mueller polarimetry to record fingermarks left on illustrated items [67]. Use of fluorescence lifetime imaging to record fingermarks left on luminescent porous substrates [68]. Assessment of four non-DSLR camera systems (i.e., intraoral camera, borescope, fibre optic camera and iPhone) to record fingermarks dusted with black powder [69] [Note: very short article; detailed results not presented.]

**Visual examinations** – Marsh et al. aimed at assessing the added value of preliminary examinations carried out before physicochemical processes [70]. In this context, the combined use of UV-A (365 nm), visible WL, and laser (445 nm or 520 nm) for latent fingermark observation is referred to as the “UVL” approach by the authors. By reviewing casework undertaken in 2020 and related to the processing of 11 motor vehicles and 15 scenes for fingermarks, the authors showed that the preliminary examinations and the physicochemical processes are complementary to each other. Indeed, from the 655 fingermarks that were observed in the above-mentioned cases, 364 (55%) were visualized with the UVL approach and 291 (45%) with the detection techniques (e.g., aluminium powder, CA fuming, NIN, protein stains, and PS). More importantly, only 4% of the recovered fingermarks were duplicated between both approaches, meaning that the preliminary examinations of items using the UVL approach led to ca. 350 fingermarks that were not detected subsequently. The efficacy of the UVL approach was especially noted for the cases involving motor vehicles, with ca. 70% of all fingermarks detected during the preliminary observations.

**Coaxial episcopy** – Williams et al. proposed the concept of a portable coaxial episcopy device that can be adapted to any camera lens [71]. The casing model was 3D-printed using black PLA filament. It was designed to combine a removable lid bearing a lens adaptor and a main casing bearing the optical components (i.e., semi-transparent mirror and diffuser). After a small-scale study involving natural fingermarks left on three flat and reflective surfaces, it was concluded that the combination of a two-way acrylic mirror with a 75/25 reflectance/transmission ratio (as a semi-transparent mirror) and frosted glass (as a diffuser) presented the best price/performance ratio. The performance of the device was then assessed by considering natural fingermarks (latent and dusted) left on six flat and reflective substrates. Overall, the coaxial episcopy outperformed diffused reflection photography.

**Full-spectrum photography** – Judd et al. investigated the added value of full spectrum imaging (i.e., UV, visible, IR) of latent fingermarks [72]. Depletion series of natural fingermarks were left on three substrates (i.e., stainless steel, soft plastic bag, and pig skin) and aged for 24 h (pig skin) or 14 days (stainless steel and plastic) before being recorded while latent. They were then dusted with a (non-forensic) UV-fluorescent powder and recorded again. Five recording conditions were considered (i.e., normal DSLR + WL, modified DSLR + WL, UV, IR/720 nm, and IR/850 nm). Each fingermark was hence recorded several times to allow a direct comparison. Eventually, the authors observed no difference between the different recording conditions and concluded that the chosen powder was not adapted to fingermark detection. Further experiments are expected.

**SWRUV imaging** – Stoddart et al. assessed the performance of UV-C imaging (aka SWRUV imaging) when recording latent and CA-processed fingermarks left on various substrates, more particularly aluminium cans [73]. In their main study, depletion series of natural fingermarks were left on cleaned aluminium cans and aged for one day to 21 days before being processed by the sequence [WL observation – SWRUV imaging – CA fuming – WL observation – SWRUV imaging – LWRUV imaging – BY40 – LUM observation]. A pseudo-operational trial was also conducted on fifteen aluminium cans collected from a recycling bin and processed as described above. For latent fingermarks, SWRUV imaging was sometimes able to suppress the background, providing added value over WL. For CA-processed fingermarks, SWRUV outperformed WL observation and LWRUV. Finally, the authors acknowledged the added value of a luminescent dye stain, with additional marks and a better contrast. The question of the impact of UV-C on touch DNA was deemed irrelevant because “In a number of operational forensic laboratories, DNA swabbing precedes fingermark detection methods” [73].

**Lifted fingermarks** – Marsh et al. proposed an alternative to the use of a flatbed scanner to record transparent lifts bearing fingermarks dusted with aluminium powder [74]. The proposed approach consists in placing the transparent lift across a slot made in a black box, illuminating it with a light source placed at a 50-degree angle, and recording the result from above. Casework-related lifts previously scanned and deemed unsuitable for comparison were recorded again using the proposed methodology. As a result, most of the fingermarks showed an increase of quality, with more than three quarters deemed suitable for comparison.

Acronyms used: **BY40** (Basic Yellow 40), **CA** (cyanoacrylate), **DNA** (deoxyribonucleic acid), **DSLR** (digital single-lens reflex), **IR** (infrared), **LED** (light-emitting diode), **LUM** (luminescence), **LWRUV** (longwave reflected UV; involving UV-A), **NIN** (ninhydrin), **PLA** (polylactic acid), **PS** (powder suspension), **SWRUV** (shortwave reflected UV; involving UV-C), **UV** (ultraviolet), **UVL** (ultraviolet, visual, laser), **WL** (white light).

#### 3.2.2 Advanced imaging

**Foreword** – Most of the papers related to advanced imaging were based on fingermarks collected in unrealistic or ideal conditions (e.g., single donor, sebum-rich or artificially-contaminated fingermarks, uncommon or pristine substrates), which are likely to result in an overestimation of the detection performances. Additionally, at the exception of a couple of papers, the position of the latent fingermarks was known beforehand. This allowed the authors to focus on a specific area without having to search the item as a whole, as in a forensic case. For these reasons, most of the cited studies are still deemed as being at a preliminary/pilot stage and were not further detailed. Additionally, three papers were not cited here-below for they deviate too much from forensic considerations.

**Preliminary/Pilot studies** – DESI-MS imaging of gelatine lifter bearing powder-dusted fingermarks to separate overlapped fingermarks [75]. TOS-SIMS imaging of latent fingermarks transferred from substrates using a transparent tape [76]. SECM imaging of sebum-rich fingermarks lifted from non-porous substrates using a PVDF membrane [77]. Separation of overlapped fingermarks left by a same donor at different times using MALDI-MS imaging [78]. TOS-SIMS imaging of latent and acid-dye-processed bloody fingermarks on porous and non-porous substrates [79]. Combination of an OCT device mounted on a robotic arm with a deep learning model to automatically locate and image latent fingermarks concealed beneath opaque adhesives [80]. DESI-MS imaging of fingermarks processed with ORO and presenting a low contrast due to the colour of the substrate [81]. Determination of the age of powder-dusted fingermarks using DESI-MS imaging [27].

**TOF-SIMS** – Three papers investigated the added value of TOF-SIMS when used in combination with conventional detection techniques [[82], [83], [84]].

In the first study, Charlton et al. aimed at improving the technological readiness of TOF-SIMS imaging [82]. Depletion series of natural fingermarks were left by two donors on two non-porous substrates (i.e., hard PE and stainless steel) and one porous substrate (i.e., office paper). After being aged for 12 to 16 days, the odd-numbered fingermarks were processed with conventional techniques (i.e., CA fuming – BY40, or NIN) while the even-numbered ones were imaged with TOF-SIMS while being latent. TOF-SIMS was also applied on fingermarks presenting too low a contrast – or not detected at all – after the application of the conventional techniques. Overall, the authors showed that fingermarks poorly detected with conventional techniques led to high quality ridge details using TOF-SIMS for all three substrates, especially on stainless steel, confirming the compatibility and the sensibility of the advanced imaging approach. As for the main limitations of TOF-SIMS, the authors emphasized the fact that it is time-consuming (i.e., ca. 37 min to scan a 6 × 6 mm area) and that some skill/knowledge are required to operate the instrument and process the data. As for the first limitation, the authors indicated that TOF-SIMS should be applied on a fingermark that has been located beforehand or partially enhanced with a conventional detection technique. In these conditions, scanning a whole fingermark (12 × 18 mm) would require ca. 3.5 h [82].

In the second study, Charlon et al. focused on two non-porous substrates (i.e., PE and stainless steel) and pursued the study described above by considering more donors, additional aging conditions (i.e., immersion in water and underground burial), longer aging times (i.e., one and five months), and an additional detection technique (i.e., Fe-BPS) [83]. Overall, high quality ridge details were obtained with TOF-SIMS imaging, regardless of the aging conditions and times. Unlike the first study, the results on PE were better than on stainless steel, which could not be explained by the authors. From a technical point of view, the authors recommend running the device in both positive and negative modes, which doubles the scanning time but maximizes the chances of imaging ridge details. Despite the very promising results, the authors enumerated the limitations of the technique: long imaging times (i.e., 2 h for vacuum, 3.5 h for a fingermark scanning), limited size of the item to be scanned (i.e., 10 × 7 cm), and necessity for the substrate to be smooth and flat (any distortion of the substrate causing an adverse effect on the imaging performance).

Similar trends were obtained by Man et al., who successfully applied TOF-SIMS on fingermarks left on various substrates (i.e., paper, aluminium, glass, plastic, and fabrics) and processed with conventional techniques (i.e., amino acid reagents, CA fuming, magnetic powder, and one-step VMD/unknown metal) [84]. The authors noted that higher quality imaging was obtained for non-porous substrates, mostly due to their surface homogeneity. [Note: despite the promising results, several lacks in the methodological section (i.e., eccrine-rich fingermarks claimed to be “natural”, and deviations – or lack of information – in the preparation and application of the conventional techniques) prevent to further discuss the relative performance of TOF-SIMS with conventional techniques from this study.]

Despite the limitations inherent to the technique, these studies offered a better understanding about how an advanced imaging technique could be introduced in an operational process, and with which added value in terms of ridge details.

Acronyms used: **BY40** (Basic Yellow 40), **CA** (cyanoacrylate), **DESI** (desorption electrospray ionisation), **Fe-BPS** (iron oxide-based black powder suspension), **MALDI** (matrix-assisted laser desorption ionisation), **MS** (mass spectrometry), **NIN** (ninhydrin), **OCT** (optical coherence tomography), **ORO** (Oil Red O), **PE** (polyethylene), **PVDF** (polyvinylidene fluoride), **SECM** (scanning electrochemical microscopy), **SIMS** (secondary ion mass spectrometry), **TOF** (time-of-flight), **VMD** (vacuum metal deposition).

### 3.3 Studies focused on detection techniques

#### 3.3.1 Amino acid reagents

**Preliminary/Pilot studies** – Monitoring of the DFO fluorescence in solution with L-glycine using different organic solvents and spectroscopic methods [85]. Proposition of a methylene-chloride-based DFO formulation [86]. Water-based hydrogels containing alloxan as a new way to detect fingermarks on porous substrates [87].

**Alternative to HFE-7100** – Due to the forthcoming phasing out of PFAS-containing carrier solvents for environmental considerations (e.g., HFE-7100 or HFE-71DE), several research projects aimed at identifying efficient alternatives for amino acid reagents [51,88,89]. The conclusions of these three studies were organized according to the alternative carrier solvents:

- Solstice® PF (Honeywell): Bouzin et al. showed no significant differences of performance between IND/Zn formulations prepared with Solstice® PF or HFE-7100, when applied to depletion series of natural (half-)fingermarks on porous substrates [88]. They however noted that the low boiling point of Solstice® PF (i.e., 19°C) implies precautions of use (e.g., solvent storage in pressurised cylinder, IND/Zn solution storage at +4°C) and handling drawbacks (e.g., IND/Zn solution to be brought back to room temperature before use, large quantities of vapour produced when processing an item, rapid evaporation of the working solution). Also, a limited shelf life and a potential detrimental impact on inks require further investigation. Able et al. performed an extensive study including depletion series of natural fingermarks and pseudo-operational trials [51]. The authors placed Solstice® PF in third position, after HFE-7100 (best) and Opteon™ SF33 (second best). Contrary to Bouzin et al., Able et al. observed no shelf-life issue, their working solutions being still performant after six months of storage at +4°C. The latest study, performed by Yergeau et al., assessed the relative performance of Solstice® PF compared to HFE-7100 for three amino acid reagents (i.e., IND/Zn, NIN, and DFO), using 6′000 natural (half-)fingermarks [89]. Their conclusions met those of Bouzin et al., which are: comparable performances for both carrier solvents, and several limitations of use for Solstice® PF (i.e., ink diffusion, storage of the solutions in pressure-resistant bottles and in a freezer, requirement to bring those back at room temperature before use). Regarding the shelf life, their conclusions met those of Able et al., with working solutions still stable after 14 months of storage in a freezer (−4 to −10°C). Despite promising performances, and considering the precautions of use described above, it must also be stressed out that Solstice® PF is a single supplier (proprietary) solvent and a PFAS by nature, as it contains HCFOs.
- Opteon™ SF33 (Opteon): Able et al. showed that Opteon™ SF33 performed similarly to HFE-7100 on depletion series of natural fingermarks and came second after HFE-7100 in the pseudo-operational trials [51]. No visible ink running was observed with NIN. However, ink running was visible under luminescence on most of the samples processed with IND/Zn using Opteon™ SF33, unlike HFE-7100. No shelf-life issues were observed, with working solutions still performant after six months of storage at +4°C. It should however be noted that Opteon™ SF33 is a single supplier (proprietary) solvent and a PFAS by nature, as it contains HFOs.
- Amolea™ AS-300 (AGC Chemicals): only non-PFAS alternative carrier solvent tested in those studies. However, Amolea™ AS-300 was shown to cause severe ink running issues, both visually and under luminescence [51]. It is also less performant than HFE-7100 and Opteon™ SF33. As a result, Amolea™ AS-300 does not represent a viable alternative to HFE-7100 [51].
- HFE-7100 (Novec, 3M): in their study, Able et al. showed that HFE-7100 was the most performant carrier solvent, mostly due to lowest level of ink running, brightest luminosity for IND/Zn, and highest number of detected fingermarks compared to the other carrier solvents [51]. However, they noted that HFE-based formulations became cloudy quicker than the other formulations when processing the items, especially for NIN, which resulted in higher quantities of solutions needed when processing items.

Despite the higher performances of HFE-7100, finding an alternative to this carrier solvent becomes critical, mostly for environmental considerations. Further studies are hence expected on this topic in the forthcoming years.

**Frugal forensic science** – With the aim of proposing an effective and sustainable IND/Zn formulation for resource limited jurisdictions, Bouzin et al. compared three formulations [90]: AFP (HFE-7100), BKA (PE) and CAST (HFE-7100). Using depletion series of natural (half-)fingermarks left on porous substrates, the authors investigated the impact of the formulations on the ridge luminescence, ridge clarity, and background staining. Overall, all three formulations presented comparable performances, with a preference for the BKA formulation, mostly due to the use of a cheap and easily available carrier solvent (i.e., PE). The authors also emphasized the need for further studies to investigate the impact of the substrates and of the polar co-solvents (i.e., ethanol/AFP-BKA, methanol/CAST) on the IND/Zn performances. The same goals were pursued by Jones et al., who assessed the use of inexpensive and household chemicals (e.g., white spirits, kerosene, Shellite, Zippo lighter fluid) as alternative carrier solvents for IND/Zn and NIN [91]. Considering depletion series of natural (half-)fingermarks, the authors concluded that (1) Shellite outperformed the other alternative solvents for IND/Zn, but was still outperformed by PE, (2) Shellite, kerosene and white spirits resulted in comparable performances for NIN. The spectrometric analyses showed that a good alternative carrier solvent should be composed of short-chained alkanes bearing minimal aromatic groups. Finally, the authors emphasized the importance of risk assessment before considering the replacement of PE with another solvent, Shellite and kerosene being carcinogenic and causing birth defects, for example.

**Donorship** – During their extensive study aiming at comparing HFE-7100 and Solstice® PF as carrier solvents for amino acid reagents (see above), Yergeau et al. also emphasized relevant trends related to donorship, depletion series, and assessor variability [89]. Regarding depletion series, fingermarks were hardly detected after the third or fourth depletion, on average.

**Processing protocol and storage conditions** – Choi et al. observed that decreasing the processing temperature for IND/Zn permitted the observation of rich fingermarks before faint marks were detected [92]. The proposed two-step protocol consists in first placing 15 sheets of paper between the item and the heat press set at 160°C, observing the results in luminescence, then processing the item again without the papers. Siem-Gorré et al. studied the impact of storage conditions on the luminescence of IND/Zn-processed items, considering printed templates of amino acid solutions and depletion series of natural fingermarks [49]. The authors observed that daylight was detrimental to IND/Zn luminescence. They concluded that the processed items should be stored in a dark environment and the detected fingermarks recorded within two days.

**Shelf life** – Shipman decided to assess the shelf life of IND and IND/Zn solutions, using spot tests made of dilution series of L-alanine [50]. The author concluded that both solutions were effective up to 30 weeks (i.e., maximum time interval tested), meaning that the commonly observed shelf life of one month could be extended.

**Hydrogel** – Clarke et al. proposed an approach based on the use of a water-based hydrogel containing IND to detect fingermarks on porous substrates [93]. Using depletion series of natural (half-)fingermarks, the authors showed that the hydrogel approach was promising but was currently outperformed by the conventional PE-based formulation.

**Drug-contaminated substrates** – Two papers investigated the impact of fingermark detection on drug-infused (porous) substrates, in the context of drug smuggling [94,95].

In the first study, Lange & Carlysle-Davies emphasized the fact that the application of NIN to substrates infused with amine-based drugs may result in ridge colours other than the expected Ruhemann’s purple [94]. Indeed, fingermarks left on substrates infused with methamphetamine, MDMA, amphetamine sulphate, and morphine appeared in dark purple/blue shades, whereas diamorphine and cocaine resulted in light grey/blue ridges. In the case of amphetamine sulphate, the substrate itself reacted with NIN and became dark purple, most likely due to the primary amines contained in the drug-infused paper. No colour variation was observed for drugs bearing ketones or amides, such as cannabinoids and barbiturates. Further developments are still needed to fully investigate the potential of using NIN as a presumptive test for drug-infused papers.

In the second study, Gulekci et al. aimed at estimating the loss of cocaine upon fingermark processing [95]. To reach that goal, the authors processed cocaine-infused paper with different fingermark detection techniques, among which several amino acid reagents (i.e., IND, DFO, NIN, ThermaNin, 5-MTN), before extracting and quantifying the remaining cocaine. [Note: IND, and not IND/Zn, was applied by the authors.] Natural fingermarks were left on office paper before and after the impregnation of the substrate with cocaine. Quite expectedly, NIN, DFO and IND led to the highest number of marks recovered. However, given that cocaine could not be retrieved from NIN-processed items, DFO or IND were hence recommended for processing such substrates. It should be noted that both reagents resulted in a loss of cocaine, estimated at −21% and −28%, respectively. [Note: these percentages were not directly reported by the authors but calculated from the concentrations reported in the paper.]

**Developed in other sections** – Impact of CA fuming on IND/Zn and NIN when processing porous and semi-porous substrates [96] – see Section 3.3.2. Comparison between DFO and IND/Zn using quality assessment algorithms as an alternative to human grading [97] – see Section 3.6.2.

Acronyms used: **AFP** (Australian Federal Police – AU), **BKA** (Bundeskriminalamt – DE), **CA** (cyanoacrylate) **CAST** (Centre for Applied Science and Technology – UK), **DFO** (1,8-diazafluoren-9-one), **HCFO** (hydrochlorofluoroolefin), **HFE** (hydrofluoroether), **HFO** (hydrofluoroolefin), **IND** (1,2-indanedione), **IND/Zn** (1,2-indanedione combined with zinc chloride), **MDMA** (3,4-methylenedioxymethamphetamine), **5-MTN** (5-methylthio ninhydrin), **NIN** (ninhydrin), **PE** (petroleum ether), **PFAS** (per- and polyfluoroalkyl substances).

#### 3.3.2 Cyanoacrylate fuming

**Preliminary/Pilot studies** – Heated mixture of DMAC, CA monomers, and octyl acetate (or methyl decanoate) as a new one-step CA fuming process [98]. Application of Diamond™ Nucleic Acid dye prior to CA fuming to emphasize the presence of DNA without preventing the detection of fingermarks by CA fuming [99]. Propositions of new dyes to be applied on CA-fumed fingermarks: DMAC combined with octyl acetate or 2-phenoxyethanol [98], europium oxide in nitric acid [100], aqueous suspension of C-dots [101], Rhodamine B hydrazones in dichloromethane [102].

**Sequential processing** – Bouwmeester conducted a study aiming at assessing the impact of CA fuming on the subsequent amino acid reagents, when applied on porous and semi-porous substrates [96]. The study consisted in three phases encompassing depletion series of so-called “groomed” and natural (half-)fingermarks deposited on various (semi-)porous substrates. For all three phases, the author emphasized that CA fuming resulted in a notable drop in performance of IND/Zn and NIN. The author eventually concluded that exposure to cyanoacrylate fumes had no significant detrimental impact on the performance of IND/Zn and NIN. [Note: this apparent contradiction may originate from the fact that if most of the detected fingermarks showed no difference between [with] and [without] CA, a detrimental impact of CA fuming was observed and illustrated for a non-negligible proportion of the deposited fingermarks throughout the study.]

**One-step CA** – Li et al. conducted a study aiming at optimizing the PCA fuming process, as well as investigating the added value of dye-staining [103]. Using thermogravimetric and differential scan calorimetry, the authors determined that the fuming temperature should be set between 212°C and 275°C. Based on depletion series of eccrine-rich (half-)fingermarks, they showed that the age of fingermarks impacted the optimal relative humidity level (i.e., the older the marks, the higher the %RH) as well as the quantity of PCA required on the heating plate (same trend). Finally, they showed that PCA presented no advantage over conventional CA in white light. The same conclusions were drawn for its inner luminescence, when compared to dye stains such as R6G when applied on compatible substrates.

**Post-processing** – Three papers addressed the question of post-processing applied to CA-processed fingermarks, with two papers proposing alternatives to existing dyes [104,105] and one paper comparing powder dusting and dye staining [106].

In the first study, Mills et al. investigated the possibility to find an alternative to RAM [104]. Considering depletion series of natural (half-)fingermarks left on various non-porous substrates (2′175 fingermarks in total), the authors showed that a 15 μM equimolar mix of R6G and MBD in methanol constituted a cheaper and more performant alternative to RAM, with an overall improvement of the quality of fingermarks on the considered substrates (i.e., +1.2% on black tape, +10.2% on plastic, +6.3% on red tape, +15.4% on clear tape, and +23.5% on foil). The authors also emphasized the impact of the substrate on the performance of CA fuming, with red electrical tape presenting the least number of detected fingermarks (∼23%) and aluminium foil the highest (∼86%).

In the second study, Gülekçi & Tülek proposed a PE-based CV formulation as an alternative to other CA dye stains (i.e., Ardrox, R6G and water-based CV) or post-processes (i.e., powder dusting) [105]. Natural fingermarks left on different non- and semi-porous substrates were considered in this study.

Finally, Stoddart et al. assessed the performance of powder dusting as CA-fuming post-process, and compared it to dye staining [106]. In the first part of the study, depletion series of natural fingermarks were left on white and semi-transparent plastic bin bags and aged for one to 28 days before being processed (20′000 fingermarks in total). Four different powders were considered (i.e., BMP, Bristol Black, *fp*Natural 1 and *fp*Natural 2) and five sequences were compared (i.e., [CA – BY40] and 4x [CA – Powder – BY40]). The results showed that the use of BY40 after CA fuming outperformed all the other sequences. Most of the powders were detrimental to fine ridge details and failed to detect any “unique” fingermark (i.e., not detected by the subsequent application of BY40). Also, even if BY40 could be applied subsequently to powders, those extended sequences were shown to be less efficient than the use of BY40 alone. In the second part of the study, a pseudo-operational trial was conducted by considering 100 plastic bags collected in supermarket recycling boxes and processed with the three most effective sequences identified during the first part. The sequence [CA – *fp*Natural 2 – BY40] resulted in the most detected fingermarks, followed by [CA – BY40]. During this operational trial, LWRUV imaging yielded a great number of CA-processed fingermarks that were not observed using WL. Altogether, the authors recommended the use of BY40 over powders as a post-process for CA fuming, unless the nature of the substrate prevents its application (i.e., semi-porous substrate). This choice was motivated by the detrimental impact powders may have on CA-processed fingermarks. If BY40 cannot be applied, BMP is to be preferred.

**Large-scale cabinet** – Barros et al. presented a large-scale CA cabinet built to process vehicles as a whole [107]. The cabinet consisted in a stainless-steel structure (i.e., 400 × 310 × 620 cm) equipped as a conventional fuming cabinet (e.g., humidity control, fans, exhaust system). The processing of an entire vehicle requires 16 heating trays (i.e., three inside and 13 around the vehicle), a total of 16 g of CA monomers and 2 h. The authors also presented three case reports, underlying the successful use of this large-scale cabinet and the detection of fingermarks on the interior parts of the vehicles (e.g., handles, levers, seat belt metallic tongue, glove compartment). On the external surfaces, more prone to dirt and moisture, the successful detection of fingermarks was more limited. Da Silva Carvalho et al. reacted to this paper by presenting another case report in which the processing of a vehicle had successfully been performed using such a cabinet [108].

**Developed in other sections** – Recording of CA-processed fingermarks using UV-C imaging [73] – see Section 3.2.1. Use of CA fuming and BY40 to detect fingermarks on immersed items [109] – see Section 3.5.2.

Acronyms used: **BMP** (black magnetic powder), **BY40** (Basic Yellow 40), **C-dot** (carbon dot), **CA** (cyanoacrylate), **CV** (crystal violet), **DMAC** (p-dimethylaminocinnamaldehyde), **IND/Zn** (1,2-indanedione combined with zinc chloride), **LWRUV** (longwave reflected ultra violet), **MBD** (7-[p-methoxybenzylamino]-4-nitrobenz-2-oxa-1,3-diazole), **NIN** (ninhydrin), **PCA** (PolyCyano UV, foster + freeman – UK), **PE** (petroleum ether), **RAM** (mix of R6G, Ardrox P133D and MBD), **R6G** (Rhodamine 6G), **%RH** (relative humidity), **UV** (ultraviolet), **WL** (white light).

#### 3.3.3 Vacuum metal deposition (VMD)

**Developed in other sections** – Use of VMD to detect fingermarks on fabrics [110,111] – See Section 3.4.4.

#### 3.3.4 Physical developer (PD)

**Developed in other sections** – Use of PD to detect fingermarks on wetted thermal papers [112] and porous items exposed to liquids [113] – See Sections 3.3.5, 3.5.2, respectively.

#### 3.3.5 Lipid stains

**Preliminary/Pilot studies** – Pre-treatment of substrates with TBAI as a new way to stabilize iodine-stained fingermarks [114]. Luminescent BODIPY dyes dissolved in a water/ethanol solution to detect fingermarks on various substrates [115].

**ORO** – Thermal vaporisation of ORO under vacuum has been proposed as a new solvent-free deposition method [116]. Considering depletion series of grease-contaminated (half-)fingermarks (i.e., wine seed oil, engine oil and Vaseline) left on various porous substrates, the authors concluded that the general performance of the gas-phase approach was inferior to the conventional one (i.e., MeOH-based ORO), especially for weaker depositions and aged marks. Harrison et al. compared two formulations of ORO (i.e., PG- and MeOH-based) with a commercially available PD formulation [112]. Considering sebum-rich and natural fingermarks left on wetted porous substrates and thermal papers, the authors emphasized the impact of the donor, of the substrate, and of the aging time on the detection performance of both ORO formulations, with an advantage for the MeOH-based one. They also emphasized the complementarity between ORO and PD, and recommended their application in sequence, starting with ORO. Finally, they noted the detrimental impact of ORO on writings, due to ink diffusion or dissolution.

**Developed in other sections** – Chemical imaging of fingermarks processed with ORO and presenting a low contrast due to the colour of the substrate [81] – see Section 3.2.2. Use of ORO to detect fingermarks on porous items exposed to liquids [113] – See Section 3.5.2.

Acronyms used: **BODIPY** (boron-dipyrromethene), **MeOH** (methanol), **ORO** (Oil Red O), **PD** (physical developer), **PG** (propylene glycol), **TBAI** (tetra-n-butylammonium iodide).

#### 3.3.6 Powder dusting

**Foreword** – As emphasized in Section 3.1.1, the total number of papers dedicated to powder dusting is 475, which represents 67% of the papers dedicated to fingermark visualization and collected for this report (n = 714). Among those, 305 papers promote the deliberate dusting of NPs, which represents 43% of the papers dedicated to fingermark visualization or 64% of the papers dedicated to powder dusting. The position of forensic researchers should be quite clear regarding the dusting of NPs, for such material could cause serious health and safety issues to practitioners through inhalation or skin contact. Some papers also promote the dusting of heavy-metal-containing particles (e.g., lead – 3 papers, cadmium – 7 papers), both extremely toxic. Finally, for most papers, fingermark detection capability was taken as a pretext for the synthesis and characterization of optically active materials – without any consideration for forensic needs, fingermark specificities, or basic methodological guidelines. For all these reasons, we decided not to cite 446 of the 475 papers dedicated to powder dusting, preventing hence the promotion of such research practices and the overload of the References Section.

**Critical review** – da Silva Carvalho et al. conducted a critical review about the use of C-dots to detect fingermarks [117]. The authors wanted to assess to which extent published studies meet fingermark detection methodology requirements, as expressed by the IFRG [2]. Out of the 85 papers that were selected, the authors emphasized a major lack of awareness from researchers with regards to forensic science knowledge and/or methodological requirements. By using predominantly fresh and sebum-rich fingermarks left on ideal substrates by a limited number of donors, combined with an absence of objective quality assessment methodology, most of the published studies were shown to be prone to bias in the way they present C-dots as an efficient fingermark detection technique.

**Preliminary/Pilot studies** – Use of black powder [118], chalk/silver powders combined with SDS [119], orange peels and rose petals [120] to dust fingermarks on items exposed to liquids [Note: in two of these studies [118,120], powder dusting was performed on porous substrates, which does not correspond to the recommended approach. The positive results were most likely due to the collection of sebum-rich fingermarks]. Buxa & Snyder showed that removing TiO_2_ from the composition of the white lanconide powder may result in a safer dusting powder, while maintaining its efficiency [121]. Promising results were obtained with (magnetic) luminescent powders made from: curcumin analogues derived from cinnamaldehyde [122], highlighter marker dyes combined with aluminium microparticles [123], fluorophores combined with chitosan, silica and sodium citrate [124]. In the context of frugal forensic science, promising results were obtained with powders made from natural compounds: lignin (extracted from rice husk) combined with thiophene chalcone [125], vitamin B2 or zinc mixed with xanthan gum or cream of tartar (potassium bitartrate) [126], roasted gram flour [127], bixin (extracted from annatto seeds) combined with kaolinite or ZnCO_3_ [128], red dye (extracted *Papaver rhoeas*, common poppies) combined with montmorillonite clay [129], natural indigo combined with kaolinite [130], grinded jamun seeds [131], tannins (extracted from black wattle) combined with bark ashes [132], pomegranate peel powder [133], commercially-available turmeric spice powders [134]. [Note: several of those papers considered the use of natural fingermarks and/or split marks for comparison with commercial powders, which constitutes a good methodological practice].

**NIR-emitting powder** – Two papers proposed the use of NIR-emitting powders to detect fingermarks on multicoloured and patterned substrates [135,136].

In the first study, Shahbazi et al. investigated the performance of exfoliated Egyptian blue particles coated with CTAB or Tween 20 [135]. Depletion series of natural (half-)fingermarks left on glass slides and (un)circulated Australian polymer banknotes were constituted to compare the performance of uncoated particles with coated ones. The authors concluded that Tween 20-coated exfoliated Egyptian blue particles were the most performant and promising.

In the second study, La Rocca et al. investigated the performance of two NIR-emitting pigments (i.e., Han blue and Han purple) [136]. Depletion series of natural (half-)fingermarks were left on various substrates, including (un)circulated Australien polymer banknotes, and aged for 48 h before being processed. Overall, Han purple performed better than Han blue and Egyptian blue. Although, the authors emphasized several background staining occurrences, mostly due to the unwanted deposition of pigment on the substrates. Further developments are hence required before promoting the use of those pigments for fingermark detection.

**Developed in other sections** – Use of powders to study fingermark aging processes [17,[19], [20], [21]] and depletion series [21] – see Sections 2.2, 2.6, respectively. Optical recording of tape-lifted fingermarks [74] – see Section 3.2.1. Relative performance of powder dusting and dye staining as CA-fuming post-processes [106] – see Section 3.3.2. Use of PS in sequence with powder dusting to improve the detection of marks on non-porous substrates [137] – see Section 3.3.7. Use of powder dusting to detect fingermarks on the outer surface of vehicles [138] or pangolin scales [139] – see Section 3.4.6.

Acronyms used: **C-dot** (carbon dot), **CA** (cyanoacrylate), **CTAB** (cetyltrimethylammonium bromide), **IFRG** (International Fingerprint Research Group), **NIR** (near infrared), **NP** (nanoparticle), **PS** (powder suspension).

#### 3.3.7 Powder suspensions (PS)

**Foreword** – The papers dealing with PS without any consideration for forensic needs, fingermark specificities, or basic fingermark detection methodological guidelines, were not cited explicitly – representing three papers.

**Fe-BPS formulation** – Fe-BPS was the main focus of three studies, one aiming at optimizing the formulation [140] and two aiming at better understanding the role played by the chemicals used in the recipes [141,142].

In the first study, Clover Lee et al. aimed at optimizing the Fe-BPS formulation before comparing it to a commercially available C-BPS (i.e., Wet Powder Black, Kjell Carlsson Innovation) [140]. For the optimization study, natural fingermarks left on different non-porous substrates and aged for 2 h were considered to assess the performance of ca. 100 Fe-BPS formulations. For the comparison study, depletion series of (half-)natural fingermarks left on the same items and aged for one day to one month were considered (ca. 3′250 fingermarks in total). [Note: the deposition process of natural fingermarks in this study deviates from the commonly encountered protocol, for the hands of the donors were washed and dried 5 min prior to each deposition – as opposed to the 30+ minutes of unsupervised activity usually reported in the literature.] The authors concluded that the powder/surfactant ratio was the main influencing factor. They retained the two most effective formulations: [Fe-BPS1] 1.7 g magnetic iron oxide (Fisher Scientific) + 1 mL [Triton X-100/ethylene glycol/water mix in a 5:7:8 ratio] + 4 mL water; [Fe-BPS2] 1.7 g iron oxide nanopowder (Sigma) + 3 mL Tween 20 4% (v/v) + 2 mL water. When comparing those formulations with the commercially available C-BPS, the authors concluded that the substrate type was the most significant influencing parameter. On plastic substrates, both Fe-BPS formulations outperformed C-BPS. On adhesive tapes, those formulations suffered from heavy background staining, although not for the same substrates, comparatively (i.e., acrylic-based adhesives for Fe-BPS1, vinyl adhesives for Fe-BPS2). Overall, the commercially available C-BPS (i.e., Wet Powder Black) was deemed the most effective and consistent.

In the second study, Clover Lee et al. aimed at better understanding the role of the surfactant in the performance of Fe-BPS [141]. To reach that goal, they collected ten different surfactants, representing the three main categories (i.e., non-ionic, anionic, and cationic). For each one, different surfactant concentrations and powder/surfactant ratios were tested. Depletion series of eccrine-rich fingermarks were deposited on three types of non-porous substrates (i.e., tile, plastic, and glass) and developed within the day of their deposition. The authors observed that all the tested formulations (even those containing no surfactant) were able to develop fingermarks, although with varying degrees of performance and background staining. Most surfactants improved the quality of the processed fingermarks (unlike the Fe-BPS formulation based on water only), the three most performant being Tween 20 (aqueous, 10% v/v), Triton X-100 (mixed with ethylene glycol and water in a 25:35:40 mL ratio), and Kodak Photo-Flo 200 (aqueous, 50% v/v). The surfactants leading to the poorest results were Tween 80 (mixed with ethanol and water in a 22:6:60 mL ratio), Span 80 + Tween 80 (mixed with water in a 5:5:90 mL ratio), and Liqui-Nox (aqueous, 25% v/v). The authors also noted that a same batch of iron oxide powder could result in variations of colour, from dark black ridges to light yellowish ones. The impact of the surfactant (type, ionic state, concentration, ratios) on the amount of powder particles (volume and size) preferentially deposited on fingermarks was discussed. The detection model, based on micelles of suspended powder particles destabilised upon contact with fingermark residue, was also debated. Overall, the authors concluded that surfactants play a crucial role in reducing unwanted deposition of particles on the substrates (background staining) and acknowledged the need for further studies on that topic.

In the last study, Maroon et al. designed a study encompassing the use of five different iron oxide powders, of varying particle sizes and providers, to better understand their role in the performance of Fe-BPS [142]. The surfactant composition (i.e., Tween 20 10% v/v) and the powder to surfactant ratio (i.e., 1:2 w/v) were kept unchanged for the study. For the optimization study, depletion series of natural (half-)fingermarks were deposited on four types of non-porous substrates (i.e., tile, plastic, glass, and adhesive tape) and aged for one day and one week before being processed. For the comparison study, half the marks were kept dry and the other half immersed in water for 30 min, before being aged for one day up to one month and processed. [Note: the deposition process of natural fingermarks in this study deviates from the commonly encountered protocol, for the hands of the donors were washed and dried 5 min prior to each deposition – as opposed to the 30+ minutes of unsupervised activity usually reported in the literature.] From the optimization study, the authors observed that the application of iron oxide in suspension outperformed powder dusting. Also, Fe-BPS made from iron (III) oxide powder (Fe_2_O_3_, Sigma-Aldrich), which is characterized by a reddish colour, outperformed all other Fe-BPS formulations made from conventional iron oxide powders (i.e., II/III). From the comparison study, the authors observed that Fe-BPS made from iron (III) oxide powder (Fe_2_O_3_, Sigma-Aldrich) outperformed the one currently recommended in the UK Home Office FVM (made from iron II/III nanopowder) on plastic and glass. On the adhesive tapes, the trend went clearly in favour of the Fe-BPS recommended in the FVM, mostly due to detrimental background staining obtained with the iron (III) oxide-based Fe-BPS.

Overall, these three studies emphasized the major impact of the PS formulation on the performances of the technique. Some key parameters related to iron oxide were also identified: manufacturer, provider, batch, and particle size. Some apparent contradictions with current recommendations or previous studies were also emphasized, which underline the need for further research in this field.

**Sequential application** – Two papers recommended the sequential application of powder dusting and Fe-BPS to improve the detection of fingermarks on non-porous substrates [137,143].

In the first study, Claveria et al. proposed to apply powder dusting and Fe-BPS in sequence, to improve the detection of fingermarks on non-porous substrates [137]. Considering depletion series of natural (half-)fingermarks left on various non-porous substrates (e.g., aluminium, glass, plastic) and aged for 45 days, the authors compared the sequence [black powder – Fe-BPS] with the application of Fe-BPS only. Overall, 55% of the high-quality fingermarks (i.e., grade 3 or 4 on the CAST scale) were detected by the combined process, compared to 42.8% for Fe-BPS alone. The only exception was observed with white plastic bags, for which Fe-BPS outperformed the combined application – without any explanation provided. It should be noted that this study constituted a way to introduce a new home-made Fe-BPS formulation, called “POSME”, made of 150 g of black powder suspended in 200 mL of a mixture containing [600 mL water, 60 mL ethanol, and 220 mL polysorbate 80], and applied by brush. Synox black 6318 was chosen for the study, due to its low price. But the authors indicated that Suspension Powder Black (BVDA – NL) and Adhesive-Side Powder Dark (Sirchie – US) performed similarly.

In the second study, Heaston replicated the above study on glass slides, PET bags, and a vehicle car door [143]. The authors replicated Claveria et al.’s methodology [Note: with the exception of using sebum-rich fingermarks, supposedly] and confirmed their conclusions.

**WET UCIO** – Horrocks et al. compared the performance of WET UCIO (i.e., a C-BPS formulation) with a NIR-emitting PS based on exfoliated Egyptian blue and a luminescent PS based on QDs in solution [144]. In the main study, depletion series of natural fingermarks were left on three adhesives type representing the three main adhesive matrixes (i.e., acrylic, synthetic and natural rubber) and aged between 24 and 76 h before being processed. In a second part of the study, donors were asked to construct IEDs from a PET bottle, adhesive tapes and a plastic box, to promote incidental depositions. Overall, WET UCIO performed the best, followed with the QD-based PS. The authors also underlined that the NIR-emitting PS based on exfoliated Egyptian blue led to inverted contrast (i.e., powder adhering to the substrate instead of the fingermarks) and resulted in the poorest detection performance. For recall, WET UCIO is made of 30 g carbon black powder suspended in 200 mL sodium lauryl sulphate (Gran Velada, Spain – conc. 27%).

**Developed in other sections** – Use of PS to detect fingermarks on adhesives [[145], [146], [147]] – see Section 3.4.2.

Acronyms used: **C-BPS** (carbon-based black powder suspension), **CAST** (Centre for Applied Science and Technology – UK), **Fe-BPS** (iron oxide-based black powder suspension), **FVM** (Fingermark Visualization Manual), **IED** (improvised explosive device), **NIR** (near infrared), **PET** (polyethylene terephthalate), **POSME** (powder suspension of Mossos d’Esquadra), **PS** (powder suspension), **QD** (quantum dot), **UCIO** (Unitat Central d’Inspeccions Oculars).

#### 3.3.8 Nanoparticles in solution

**Foreword** – The papers dealing with NPs in solution without any consideration for forensic needs, fingermark specificities, or basic fingermark detection methodological guidelines, were not cited explicitly – representing 15 papers.

**Preliminary/Pilot studies** – Different NPs suspended in aqueous solution were proposed to detect fingermarks on non-porous substrates: fluoride NPs [148], amino-functionalized poly (*p*-phenylenevinylene) NPs [149], aqueous suspension of C-dots to be used as a CA dye [101], ZnO NPs mixed with diethylene glycol monoethyl ether to be used as an SPR on wetted items [150], and Eu-Tb MOF NPs in aqueous solution to detect fingermarks on porous substrates [151]. [Note: about this last paper, despite the huge number of fingermarks claimed to have been deposited by 173 donors (i.e., 9′600) on several substrates (i.e., porous, non-porous, semi-porous) and aged up to 150 days, major lacks in the described methodology make it difficult to properly assess the actual performance of the proposed reagent. Among others: lacunar information about the considered substrates and about the fingermark deposition process, no information about the formulations and application protocols of the techniques used as references, such as IND and a commercial blood reagent).]

**Developed in other sections** – Use of QDs in solution to detect fingermarks on adhesive tapes [144] – see Section 3.3.7. Use of natural lipase and carbon nanotubes to detect fingermarks on wetted items [152] – see Section 3.5.2.

Acronyms used: **CA** (cyanoacrylate), **C-dot** (carbon dot), **IND** (1,2-indanedione), **MOF** (metal-organic framework), **NP** (nanoparticle), **SPR** (small particle reagent).

#### 3.3.9 RECOVER LFT

**Preliminary/Pilot studies** – Sequential use of gelatine lifting and RECOVER LFT on porous substrates to assess whether a fingermark lies above or below a printed text [153].

**Developed in other sections** – Several papers aimed at assessing the performance of the RECOVER LFT device to detect fingermarks on metal surfaces [[154], [155], [156], [157], [158]] – see Section 3.4.1.

#### 3.3.10 Other detection techniques

**Foreword** – The papers dealing with new reagents without any consideration for forensic needs, fingermark specificities, or basic fingermark detection methodological guidelines, were not cited explicitly – representing 64 papers. Among those, are included ca. 55 papers dedicated to AIE-capable reagents, which were published during the period covered by this report (as opposed to a dozen only, for the previous report [1]). Despite the increased number of papers, the use of compounds presenting AIE capabilities to detect fingermarks remains at a preliminary stage, mostly due to the use of fresh, sebum-rich fingermarks left by a very limited number of donors (for most of them: only one) on ideal substrates.

**Preliminary/Pilot studies** – Use of a wet nitrocellulose membrane, doped with ink and chitosan, to lift fingermarks from non-porous, semi-porous and porous substrates [159]. Spraying of imidazolinone-based fluorescent dyes in aqueous solution to detect sebum-rich fingermarks on various substrates [160]. Use of a cellulose-based film, containing silicon dioxide NPs functionalized with nitrile groups, to lift fingermarks from non-porous substrates [161]. Immunodetection of keratin and dermcidin (contained in fingermarks) combined with a biotin-avidin labelling system [162].

**Case report** – Radford reported the case of fingermarks left on the exterior side of a car window and made visible by the morning dew [163].

**Iodine** – Clarke et al. proposed to apply a 4% (w/w) starch hydrogel on iodine-fumed fingermarks left on porous substrates to fix the colouration [164]. Considering natural fingermarks left on three types of paper (i.e., starch free, copy, and recycled copy) and aged for one day up to seven days (720 fingermarks in total), the authors compared their approach with an all-in-one iodine-benzoflavone solution. The results showed that the benzoflavone solution outperformed the hydrogel. The authors acknowledged that further research are required.

**Historical considerations** – Claveria & Delgado presented five detection techniques documented before 1880, namely: iodine fuming combined with silver nitrate-acetate (Paul-Jean Coulier), silver nitrate, mercury protonitrate, Pointevin liquid (i.e., iron perchloride and tartaric acid) combined with palladium chloride (1874, Pierre François Aubert), and osmic acid (Adrien Charpy) [165].

**Reviews** – Assessment of various solvent-free approaches to detect fingermarks on paper [166]. Of the three overviewed approaches (i.e., chemical fuming, dry transfer, and application of heat), dry transfer was identified as being the most promising one for implementation.

Acronyms used: **AIE** (aggregation-induced emission), **NP** (nanoparticle).

### 3.4 Studies focused on substrates

#### 3.4.1 Metal and cartridge cases

**Preliminary/Pilot studies** – Electrochromic detection of fingermarks on stainless steel using manganese oxide [167] or a xanthan hydrogel containing a chromophore [168]. Detection of lipid-enriched fingermarks on various metals by electrochemical deposition of copper NPs [169], silver NPs [170], and polyphenazine polymers [171,172]. Detection of sebum- and/or eccrine-rich fingermarks on brass by electrodeposition of nickel [173] or phenotiazine co-polymers [174]. Detection of sebum-rich fingermarks on stainless steel by electroplating of silver and copper [175]. Combination of octyl-CA fuming and Pd electroless deposition to promote the reverse development of sebum-rich fingermarks on unfired brass ammunitions exposed to outdoor conditions [176].

**RECOVER LFT** – Five papers aimed at assessing the performances of the RECOVER LFT device (foster + freeman – UK) to detect fingermarks on (un)fired cartridge cases [[154], [155], [156]], stainless-steel knives [157], or metal plates [158].

First, Exall et al. conducted an extensive pseudo-operational study focused on the most encountered ammunition types in UK [154]. Their study was based on natural fingermarks deposited on unfired and fired (nickel-plated) brass cartridges for semi-automatic pistol (mostly), and on some larger cartridges (e.g., military type). The fingermark deposition scenario consisted in loading handgun magazines with seven to ten rounds in a natural way. After different aging times, the ammunitions were either discharged (Experiments 1 and 3) or removed manually from the magazines (Experiment 2), before being processed by RECOVER LFT or CA fuming (combined with BY40). In total, over 1′000 ammunitions/cartridge cases were processed. Overall, the detection performance was extremely low for all the techniques. In the first two experiments, neither RECOVER LFT nor CA fuming successfully detected quality fingermarks (i.e., grade 3 or 4 on the CAST scale) on fired and unfired ammunitions. In the last experiment (based on ca. 250 fired cartridge cases per technique), the recovery rate for Grade 3 marks was only of 1 – 2%. The authors concluded that both RECOVER LFT and the sequence [CA fuming – BY40] resulted in comparable performances (although low), and that further optimization studies are required to properly use the RECOVER LFT.

In the second study, Lam et al. aimed at better understanding the proper way to use the RECOVER LFT [155]. To reach that goal, they first considered natural fingermarks left on brass ammunition (ca. 1′500 fingermarks in total). The fingermark deposition scenario consisted in holding the cartridge for 3 s before placing it back on the holder. This way of doing allowed the authors to constitute depletions series by asking a donor to manipulate several cartridges in a row. Some ammunitions were then loaded into magazines, some of them being discharged and others ejected without being fired, while the remaining ones were kept unloaded. Multiple detection techniques, sequences, and cleaning protocols (prior to RECOVER LFT) were then tested on those cartridges. A pseudo-operational trial has also been conducted with 1′000 fired brass ammunition collected from different shooting ranges and processed with RECOVER LFT with (500) and without (500) a prior-washing step. Given the size of this study, interested readers are redirected to the original paper for fully detailed results and a complete overview of the observations made by the authors. Only a selection of the observations is presented here-after: (i) for unfired ammunitions, the cleaning step should be omitted and the RECOVER LFT process should be shorter than for fired ones, (ii) the application of RECOVER LFT after the sequence [CA fuming – BY40] or in sequence with VMD_Au/Zn_ or VMD_Ag_ resulted in added value (e.g., quality improvement or additional marks), (iii) in the pseudo-operational trial, an evidence of touch has been emphasized for 37% of the cartridges, but fingermarks suitable for comparison were retrieved on 1.6% (no prior-washing step) and 2.0% (prior-washing step) of them only. The authors also detailed the issues encountered with the RECOVER LFT device (e.g., hardware and software troubleshooting, improper precursor) and the ways to address those.

In the third study dedicated to ammunitions, Wong et al. investigated if fingermarks processed with RECOVER LFT do continue to develop over time [156]. To reach that goal, sebum-rich fingermarks were deposited by a single donor on pre-cleaned brass cartridges, which were then fired after 1 h or one day of aging. The authors observed an increase of quality for ca. 20 – 30% of the detected fingermarks, from 48 h up to seven days after having been processed, resulting in fingermarks becoming “suitable for identification”. During the same period, some fingermarks underwent a decrease of quality. The authors concluded that the observation should be performed as soon as possible after the RECOVER LFT process and re-conducted several days after. The authors finally discussed the limitations of their methodology, as well as some hardware issues encountered with the device.

In their study, Kirk et al. assessed the performance of the RECOVER LFT to detect fingermarks on the blade of stainless-steel knives [157]. Depletion series of natural fingermarks were left on the blade of four brands of knives, for a total of ca. 3′000 fingermarks which were aged from one day to four months. The performance study consisted in comparing the stand-alone performance of RECOVER LFT relatively to four conventional processes (i.e., CA fuming + BY40, C-BPS, BV3, and AY7), as well as when applied in sequence to those. Overall, the authors observed that the RECOVER LFT performed similarly to C-BPS and outperformed the other techniques. It was also able to detect additional fingermarks, or enhance the quality of detected ones, when applied in sequence. The most performant combination was the one involving C-BPS followed with RECOVER LFT. All other sequences resulted in less fingermarks than when the RECOVER LFT was used as a stand-alone process. Other observations that were made about the RECOVER LFT (i) the prewash step (i.e., soapy water and clean cloth): is not recommended as it led to a 50% drop in performance, (ii) a sub-optimal vapour circulation into the chamber was emphasized for large items, and (iii) the detection mechanism involving corrosion was questioned. Finally, the authors emphasized a loss of contrast with time on processed items and hence recommended to record the fingermarks within 24 h of processing.

Craven et al. conducted different experiments aiming at assessing the impact of the secretions, donorship, aging time, prewash prior processing, as well as of the contact with a corrosive substance and the integration into an existing detection sequence [158]. Overall, (depletion series of) eccrine-rich and sebum-rich (half-)fingermarks were deposited on pre-cleaned brass and stainless-steel plates. The fingermarks were then aged from 10 min up to six months (depending on the experiment) before being processed with the RECOVER LFT. Given the size of this study, interested readers are redirected to the original paper for fully detailed results and a complete overview of the observations made by the authors. Only a selection of the observations is presented here-after: (i) sebum-rich fingermarks resulted in higher detection performance compared to eccrine-rich ones, (ii) prior-washing step is not recommended for metal plates, which is in agreement with Lam et al.’s conclusions about unfired cartridges, (iii) bleach-exposed fingermarks could still be detected, and (iv) the RECOVER LFT process slightly outperformed the sequence [CA fuming – R6G – powder dusting], mostly due the collection of sebum-rich fingermarks.

Overall, these five studies showed that the mechanisms behind the RECOVER LFT remain poorly understood by the community, and people are still trying to determine a proper way of using this device. The impact of the prior-washing step remains unclear, even if an agreement seems to emerge towards a detrimental impact of this step when the item to process is not a fired cartridge [155,157,158].

**Developed in other sections** – Impact of the firing event on the composition in lipids of fingermarks left on cartridges [4] – see Section 2.2. Impact of heat on the detection of fingermarks on stainless steel and tinplate [177] – see Section 3.5.3.

Acronyms used: **AY7** (Acid Yellow 7), **BV3** (Basic Violet 3), **BY40** (Basic Yellow 40), **C-BPS** (carbon-based powder suspension), **CA** (cyanoacrylate), **NP** (nanoparticle), **R6G** (Rhodamine 6G), **VMD** (vacuum metal deposition).

#### 3.4.2 Adhesives and tapes

**Transfer phenomenon** – Four papers investigated the transfer of fingermarks from – or towards – adhesives [[178], [179], [180], [181]].

In the first study, Anderson investigated the transfer of fingermarks between overlapping duct tapes [178]. Using fresh sebum-rich fingermarks provided by two donors, the author observed that the transfer of fingermarks occurred in both directions (from/towards the adhesive side) regardless of the tape quality (i.e., expensive or inexpensive), the transfer time (i.e., one week to three months) or the separation method (i.e., freezing, adhesive remover, or manual).

In the second study, Draxel et al. investigated the transfer of fingermarks from two non-porous substrates (i.e., aluminium and plastic sheet) towards the adhesive side of duct tapes, as well as between two overlapping duct tapes [179]. Similarly to Anderson, fresh sebum-rich fingermarks were considered in this study, with additional considerations such as depletion series and half-marks. Contrarily to Anderson’s study, the authors observed no transfer of fingermarks from/towards the adhesive side after a one-week transfer period. They did however observe a transfer of fingermarks between two overlapping tapes, in the case of a good donor and a 30-min transfer time.

In those first two studies, the authors recommended to take into consideration the possibility of a transfer when processing (overlapping) tapes, especially because the observed pattern would be horizontally flipped compared to the original one.

In the third study, Recker focused on the possibility to transfer a latent fingermark from one surface to another, using Scotch® tape, for forgery purposes [180]. Using fresh sebum-rich fingermarks deposited on a clear drinking glass, the author demonstrated the possibility of a transfer towards a knife blade by using classic tape and vigorously rubbing the non-adhesive side. The transferred fingermarks were either visible as latent or after powder dusting. The author somewhat noted the presence of a halo around the transferred fingermark, presenting no dusting background and supposedly caused by the tape-transfer.

Finally, Aronson et al. investigated the possibility for ridge details to be transferred when wearing latex gloves and manipulating pressure sensitive adhesives [181]. Donors were asked to wear natural rubber disposable latex gloves between 10 and 30 min before touching the adhesive side of different adhesives (e.g., electrical, box-sealing, double sided, duct). Uncharged and sebum-enriched fingertips were considered in this study. The authors observed that good quality ridge details could pass through latex gloves and be transferred towards three of the tested adhesives (i.e., Scotch® Vinyl Electrical tape 35, Scotch® Magic TM tape 810, and Tartan™ General Purpose Box Sealing Tape). The other adhesives showed no sign of such a transfer, supposedly due to their surface texture. Also, the transfer mechanism seemed to be driven by physical considerations rather than by a transfer of secretions. The authors also observed a difference of behaviour when processing the adhesives with BPS. Contrarily to fingermarks created by the direct contact of a fingertip with the adhesive side, the fingermarks transferred through glove showed no deposition of powder on the ridges, leading to a tonal reversal phenomenon (i.e., lighter ridges over a darker background).

**Powder suspensions** – Three papers were dedicated to the optimization of BPS formulations to detect fingermarks on the adhesive side of pressure-sensitive adhesive tapes [[145], [146], [147]].

First, Schwartz et al. investigated if a modification of pH could impact the performance of the C-BPS used in Germany [145]. Considering depletion series of natural (half-)fingermarks left on three general types of tapes (i.e., water-based acrylic, natural rubber-based, mixed material-based), the authors showed that a pH value between 3 and 4 resulted in higher quality marks, with no background staining, compared to lower or higher pH values. An optimized C-BPS formulation was hence proposed: 150 mg of citric acid monohydrate dissolved in 90 mL of deionised water, with 10 g of sodium lauryl ether sulphate (70%, water-based) and 7 g of carbon-black powder.

Bouzin et al. investigated the possibility to modify the original WET UCIO formulation so that it meets frugal forensic science requirements [146]. Their study relied on depletion series of natural (half-)fingermarks deposited on 12 different tapes, distributed into four main categories (i.e., packaging, masking, insulating, and duct tapes). The authors showed that the commercial Gran Velada 27% (w/v) SDS surfactant, recommended for the WET UCIO, can be replaced with 15% (w/v) SDS in 5% (v/v) ethanol/deionised water. Consequently, the new BPS formulation (called “Wet SPF”) proved to be at least as effective as WET UCIO and a commercial BPS (i.e., Wetwop™). This study also emphasized the impact of the surfactant concentration on the performance of BPS, too low a concentration leading to unwanted background staining. In terms of shelf life, the Wet SPF solution remained effective over six months. Finally, it should be noted that the authors demonstrated the possibility to apply BPS on a black insulating tape, by taking advantage of the difference in luminescence between the BPS-processed fingermark (luminescent) and the tape surface (darker).

The same objectives were targeted by Pitman et al., who considered household products (e.g., shampoos, dishwashing liquid, laundry wash) as replacement for the SDS [147]. Some positive results were obtained, but the study was extremely preliminary, and further experiments are required before emitting valid conclusions.

**Developed in other sections** – Impact of solvent-based adhesive removers on the subsequent analysis of touch DNA [182] – see Section 3.7.1.

Acronyms used: **BPS** (black powder suspension), **C-BPS** (carbon-based powder suspension), **SDS** (sodium dodecyl sulphate), **UCIO** (Unitat Central d’Inspeccions Oculars).

#### 3.4.3 Banknotes

**Processing of £10 and £5 banknotes** – Joannidis et al. presented a pseudo-operational trial aiming at comparing two detection sequences (determined to be the most effective in a previous study) on £5 and £10 banknotes issued by the Clydesdale Bank and the Royal Bank of Scotland [183]. The compared sequences were: [PCA – BMP] and [Fe-BPS]. The pseudo-operational trial was conducted over a four-week period during which 204 banknotes (ca. 20% uncirculated and ca. 80% circulated) were left for random handling by the laboratory staff before being processed. The authors concluded that the sequence [PCA – BMP] was the most effective, with 84% of banknotes presenting useable ridge details. The second sequence followed closely, though, with 78% of banknotes. The authors also observed a difference in performance between: (i) the issuing banks, in favour of Clydesdale Bank, (ii) the denomination of the banknote, in clear favour of £10, and (iii) the uncirculated and circulated banknotes, in clear favour of the uncirculated ones. The latter observation was consistent with the conclusions of the study performed by Jones et al. [184] and described here-below. Finally, the authors recommended the use of IR light source (i.e., 730-800 nm combined with an 815 nm filter) to reduce background interference.

**Impact of handling** – Jones et al. investigated the impact of polymer banknotes handling on the recovery of fingermarks [184]. For this study, the authors considered new and handled £5 banknotes issued from four different banks, three detection techniques (i.e., CA fuming + BY40, VMD_Au/Zn_, and magnetic fluorescent powder), and ca. 2′000 deposited natural fingermarks. The handling protocol consisted in a manual handling of new banknotes for 2 min, to mimic standard circulation (e.g., folding and crumpling). The end-of-life state was mimicked by applying the handling protocol five times, interspersed with one-day-long storage on an individual. Surface degradations upon handling, such as roughness increase, surface cracking or intaglio printing loss, were observed by AFM and LSCM. On the Clydesdale Bank £5 banknotes, the authors concluded that VMD_Au/Zn_ outperformed powder dusting. When considering all four issuing banks, both VMD_Au/Zn_ and [CA fuming + BY40] were equally performant. Throughout the study, the authors emphasized the detrimental impact of banknote handling on the detection effectiveness. The decrease in detection performance was however less pronounced for VMD_Au/Zn_ compared to CA fuming and powder dusting, both heavily impacted.

Acronyms used: **AFM** (atomic force microscopy), **BMP** (black magnetic powder), **BY40** (Basic Yellow 40), **CA** (cyanoacrylate), **Fe-BPS** (iron oxide-based black powder suspension), **IR** (infrared), **LSCM** (laser scanning confocal microscopy), **PCA** (PolyCyano UV, foster + freeman – UK), **VMD** (vacuum metal deposition).

#### 3.4.4 Fabrics

**VMD** – Horvath proposed to determine the best metal configuration to detect fingermarks on fabrics using VMD [110]. Twelve different fabrics were considered: three synthetic (i.e., felt, polyester, and satin) and three natural ones (i.e., cotton, linen, and denim), each represented in dark and light colours. Regarding VMD, eight metal configurations were tested: three mono-metallic (i.e., Ag, Ag sterling, and Cu) and five bi-metallic (i.e., Au/Zn, Ag/Zn, Al/Zn, Ag_sterling_/Zn, and Cu/Zn). Based on ca. 3′000 natural fingermarks provided by seven donors, the study showed that the best-so-far performances were obtained with tight-weave and high thread count fabrics (e.g., satin, polyester, denim), and by the bimetallic VMD configurations. On that matter, Cu/Zn and Al/Zn provided the best-so-far results. [Note: the use of “best-so-far” is justified by the fact that, overall, most fingermarks were not detected or were of the lowest quality grade, emphasizing the difficulty to detect fingermarks on fabrics.] Finally, the detection performances dropped for all substrates as the fingermarks aged, with almost no quality marks obtained after 28 days. Kent reacted to this paper by regretting the absence of any image of detected fingermarks and by discussing a misleading interpretation of the UK Home Office FVM regarding VMD applied to fabrics [111]. He finally emphasized that fabrics remain an extremely difficult substrate to detect fingermarks on, with very limited operational success.

Acronyms used: **FVM** (Fingermark Visualization Manual), **VMD** (vacuum metal deposition).

#### 3.4.5 Thermal papers

**Preliminary/Pilot studies** – Proposition of a DFO formulation containing PVP and Au/Ag core-shell NPs to detect fingermarks on thermal papers [185].

**Acetone removal process** – Styx & Brown investigated the performance of acetone removal when applied before (pre-processing) or after (post-processing) IND/Zn and NIN [57]. Overall, it was concluded that the process was effective to remove the thermal layer and improve the quality of the detected fingermarks. Some exceptions were underlined, such as a major detrimental effect on thermal labels and a few instances of degraded ridge patterns on thermal receipts. The authors also observed that the acetone removal process had no detrimental impact on nonthermal substrates, meaning that no adverse effect is to be expected in case of doubt. Also, the authors recommended the use of acetone as a pre-processing step whenever possible, although it was equally efficient as a post-processing treatment. It should be noted that the study was performed on (half-)fingermarks enriched in artificial secretions (i.e., Latent Print Standards Pad, Sirchie – US). The authors justified this choice to focus the study on the acetone removal process, but they eventually concluded that additional research encompassing natural fingermarks were needed.

**Developed in other sections** – Performance of ORO and PD on wetted thermal papers [112] – See Section 3.3.5. Use of thermal papers to transfer fingermarks from the skin of dead and living bodies [186] – See Section 3.4.6.

Acronyms used: **DFO** (1,8-diazafluoren-9-one), **IND/Zn** (1,2-indanedione combined with zinc chloride), **NIN** (ninhydrin), **NP** (nanoparticle), **ORO** (Oil Red O), **PD** (physical developer), **PVP** (polyvinylpyrrolidone).

#### 3.4.6 Other substrates

**Preliminary/Pilot studies** – Sequential application of CA fuming and silicone casting to detect and collect fingermarks left in/with anti-climb paint [187].

**Biodegradable/Compostable plastics** – Two studies addressed the question of fingermark detection on biodegradable and/or compostable plastics [188,189].

In the first study, Illston-Baggs et al. considered 13 “eco-friendly” soft plastic materials, several detection techniques (e.g., CA fuming + BY40, powder dusting, Fe-BPS, NIN, VMD) applied alone or in sequence, and depletion series of natural fingermarks [188]. Overall, the conclusions were the following: (i) no single sequence suited all eco-friendly plastics, (ii) the best performance was obtained with the sequence [CA fuming – BY40 – VMD_Au/Zn_ – VMD_Ag_], which is similar to the one recommended by the UK Home Office FVM for soft plastic packaging, and (iii) fpNatural powders, NIN, and Fe-BPS were the least performant techniques overall. It should be noted that the sequence presented in (ii) was the most effective by far on the compostable carrier bags currently in use in UK supermarkets, which is the country the study was carried out. About CA fuming, the results showed that one-step fluorescent CA (i.e., LCA and PCA) provided no added value compared to conventional CA, with a very weak intrinsic luminescence – if observed at all. Finally, the authors provided a description of the behaviour of the different plastics when being processed (e.g., shrinkage, change of texture), which may be helpful for people having to process such substrates.

In the second study, Woodward et al. considered six types of “environmentally friendly” plastics – each representing a main category (e.g., bio-PE, starch-based compostable, rigid PLA), four detection techniques (i.e., CA fuming, VMD_Au/Zn_, C-BPS/Ti-WPS, and SMD II), and depletion series of natural (half-)fingermarks (ca. 6′500 fingermarks in total) [189]. [Note: the deposition process of natural fingermarks in this study deviates from the commonly encountered protocol, for the hands of the donors were washed and dried 5 min prior to each deposition – as opposed to the 30+ minutes of unsupervised activity usually reported in the literature.] Overall, the conclusions were the following: (i) the performance of each technique is strongly related to the nature of the plastics (e.g., CA fuming performing well on PE + but extremely poorly on starch-based plastics), (ii) not considering PE + plastics, the most consistent and effective methods were SMD II, followed with PS and CA fuming, and (iii) VMD_Au/Zn_ was the least performant technique overall.

Both studies share common conclusions, such as a strong impact of the types of biodegradable/compostable plastic on the fingermark recovery process. But they also differ with regards to the performances of the investigated techniques, such as for PS and VMD. Differences in methodological choices between both studies most likely explain those divergences: types of biodegradable/compostable plastics, composition of the PS (i.e., Fe-BPS for the first study; C-BPS and Ti-WPS for the second one), application of the detection techniques (i.e., in sequence for the first study; as stand-alone for the second one), and quality assessment (i.e., absolute grading for the first study; relative grading for the second one).

**Sticky notes** – The possibility of a transfer between stick notes and the underlying substrates was investigated by Croxton et al. [190]. Depletion series of natural fingermarks were left on two types of sticky notes, in a way to span both the adhesive and the non-adhesive areas. Thirty minutes after, the sticky notes were stuck on different porous substrates, and a weight (5 kg) was placed on top. The setup was left as such from two to 72 h before both items (i.e., the sticky note and the underlying substrate) were processed with IND/Zn. The authors demonstrated that a transfer could occur, mostly from the adhesive area of the sticky notes, but the quality of the transferred fingermarks were poor and dependant of the paper type. They also emphasized the possibility to collect a fingermark from a non-porous substrate (i.e., glass slide) using a sticky note, then transferring it towards another surface. However, the procedure requires the same setup (i.e., fresh fingermarks, 5 kg weight, hours-long transfer), making this process less likely to occur in real cases.

**Skin** – Gülekçi et al. studied the transfer of fingermarks from the skin of living and dead bodies to thermal papers, before processing those with ThermaNin or BMP [186]. Natural fingermarks were left on smooth skin areas (i.e., forehead, neck, and wrist) and aged between one and 5 h before being transferred. After having located the fingermarks on the skin using a 315-400 nm UV light source, the authors placed a thermal paper over the area of interest and rolled a 500 g metal cylinder to promote the transfer. They repeated this step with another thermal paper to transfer the remaining material. The authors concluded that ThermaNin was more performant than BMP to detect the transferred marks. Also, a decrease of performance was observed as the fingermarks aged, also between living bodies and deceased ones.

**Gloves** – Rajs et al. investigated the detection of fingermarks on the inner side of disposable latex gloves [191]. Considering gloves worn for 10 to 15 min before being removed, the fingermarks were processed after being stored for 12 days with different detection techniques (i.e., CA fuming, CV as dye stain, and NIN) applied in sequence. The sequence [NIN – CA fuming – CV] led to the highest number of quality marks detected (i.e., ca. 30%). Gee proposed a review dedicated to the transfer of chemical components from gloves to surfaces when items are handled, based on occurrences witnessed in medicine, food and material sciences [192]. With this review, the author aimed at raising awareness for glove marks, and their potential impact or interest for forensic science.

**Glassine stamp bags** – Barnes et al. investigated the best way to detect fingermarks on glassine stamp bags, a semi-porous substrate usually encountered in heroin packaging cases [193]. Natural fingermarks were aged up to 12 months before being processed with four detection techniques (i.e., magnetic powder, DFO, and NIN). The results showed that magnetic powder was the most performant method for fingermarks aged up to three months, whereas DFO was most performant for older fingermarks. The authors also emphasized a phenomenon of glove transfer occurring when handling the items.

**Vehicles** – Wang & George assessed the performance of liquid latex applied with a foam roller on the exterior surface of vehicles, as a pre-treatment for fingermark recovery with powder dusting [138]. To reach that goal, sebum-rich fingermarks were left by a single donor on the lower areas of cleaned vehicles, which were then driven during two to four weeks before being processed for fingermarks. The study showed that the liquid latex pre-treatment was detrimental to fingermark recovery, with less marks detected compared to the direct application of powder. These conclusions contradicted the promising results of the preliminary study conducted by the authors and described in the previous Interpol report [1]. The authors explained those differences by the time of the year the experiments were conducted (i.e., winter vs summer) and a significant difference of debris accumulated on the vehicles between both studies.

**Silver mirrors** – Accioly et al. investigated the performance of mirror delamination (combined with dark field transmitted lighting) when compared to conventional detection techniques (i.e., black powder dusting + lifting and CA fuming) and visualization modes (i.e., polarized light) [194]. For recall, the delamination process consists in removing the back side of mirrors, using a combination of paint remover gel and diluted nitric acid, to get a transparent and non-reflective substrate. Considering depletion series of natural fingermarks left on mirrors, the authors concluded that the delamination process was more performant than [(CA fuming) – black powder dusting – lifting] but less performant than preliminary observations using polarized light. Overall, it was concluded that mirror delamination (combined with dark field) could constitute a good alternative to polarized light and be combined with conventional fingermark detection techniques, applied subsequently.

**Wildlife** – Woodcock et al. conducted a study aiming at determining the best combination of powder and lifter to detect fingermarks on pangolin scales [139]. Natural fingermarks were left on pangolin scales before being processed with different kinds of powders (e.g., regular, nanoscale, non-luminescent, luminescent, metallic, C-based). Conventional aluminium, magneta flake, and red fluorescent powders were identified as being the most effective ones. Although, a significant loss of performance was observed as the fingermarks aged, from 1 h to seven days. With regards to lifters, no significant differences were observed between the black gelatine lifter and tape lifters, all behaving similarly up to 50°C room temperature. Woodcock et al. also published a review about fingermark detection on wildlife animals (e.g., pangolin scales, bird feathers, eggs, ivory, leather) [195]. The authors emphasized the efficiency of common fingermark detection techniques for this purpose and discussed the limitations of the reported methodologies.

**Developed in other sections** – Impact of airflow dust deposition on the quality of fingermarks left on mirrors [196] – See Section 3.5.4. Impact of fingermark detection techniques on the isotope ratio analysis of PE films [197] – See Section 3.7.1.

Acronyms used: **BMP** (black magnetic powder), **BY40** (Basic Yellow 40), **CA** (cyanoacrylate), **C-BPS** (carbon-based black powder suspension), **CV** (crystal violet), **DFO** (1,8-diazafluoren-9-one), **Fe-BPS** (iron oxide-based black powder suspension), **FVM** (Fingermark Visualization Manual), **IND/Zn** (1,2-indanedione combined with zinc chloride), **LCA** (Lumicyano, Crime Science Technology – FR), **NIN** (ninhydrin), **PCA** (PolyCyano UV, foster + freeman – UK), **PE** (polyethylene), **PE+** (polyethylene with degradable additives), **PLA** (polylactic acid), **PS** (powder suspension), **SMD** (single metal deposition), **Ti-WPS** (titanium dioxide-based white powder suspension), **UV** (ultraviolet), **VMD** (vacuum metal deposition).

### 3.5 Studies focused on contextual situations

#### 3.5.1 Blood-containing fingermarks

**Preliminary/Pilot studies** – Use of an aqueous solution of sulfonate indolizine squaraine, a NIR fluorescent dye presenting a “turn-on” mechanism upon binding with serum albumin [198]; Promising results were obtained with diluted and scrubbed bloodstains left for aging up to seven days. Exposition of bloody marks (i.e., foot- and shoe-) left on floor tiles to a heating process (i.e., 200°C for five to 10 min) as an alternative to conventional fixing solutions (e.g., 5-SSA) before the application of a protein stain [199]; Promising results were obtained but the approach currently suffers from the fact that the optimal heating time depends on the quantity of blood, resulting in cases of local under- and over-heating leading to blood leaching and improper protein staining, respectively.

**Protein stains** – Two studies proposed to use a combined fixative/stain approach for the processing of bloody fingermarks with aqueous-based protein stains: AB [200] and HR [201].

In the first study, the combined approach consisted in merging the conventional fixative and staining solutions by solubilizing the right quantity of 5-SSA directly into the WEAA-based staining solution [200]. For the relative performance assessment, bloody (half-)fingermarks were left on substrates that could be encountered in casework (i.e., painted gyprock, vinyl tile, and varnished wood) and aged from one day to one month before being processed with either the combined or the multi-step AB process. Overall, the results showed that both processes performed similarly for most of the marks (63.5%), with the remaining corresponding half-marks either in favour of the combined approach (23.7%) or of the multi-step one (11.3%). The authors noted that the combined AB formulation led to slightly darker ridges, compared to the conventional formulation. Also, in both cases, a rinsing step was still required.

In the second study, the approach was similar in substance but deviated with regards to the composition of the combined approach, which was not based on the WEAA formulation. Ultimately, the combined HR formulation proposed by the authors consisted in dissolving 20 g of 5-SSA and 2 g of HR in 1L of deionised water [201]. For the relative performance assessment, depletion series of (half-)fingermarks were deposited on black and white tiles (ca. 3′000 fingermarks in total) and processed with different protein stains, including the combined AB formulation presented above. Additional tests were performed on substrates that could be encountered in casework (i.e., painted wood and plasterboard, metal sheets and vinyl flooring), and aimed at comparing the performance of the proposed formulation with a commercially available multi-step HR formulation. Overall, the authors concluded that the combined HR formulation performed similarly as (or better than) the other protein stains (i.e., WEAA-based AY7, combined AB, commercial HR).

**Reviews** – Extensive review about the current detection techniques, and the recent developments, applied to bloody fingermarks [202].

**Developed in other sections** – Age estimation of bloodstains using electrochemistry [24,25] – See Section 2.2.

Acronyms used: **5-SSA** (5-sulfosalicylic acid), **AB** (Amido Black), **AY7** (Acid Yellow 7), **HR** (Hungarian Red; synonym: Acid Fuchsin), **NIR** (near infrared), **WEAA** (water – ethanol – acetic acid).

#### 3.5.2 Items exposed to liquids

**Preliminary/Pilot studies** – Use of ZnO NPs mixed with diethylene glycol monoethyl ether to detect fingermarks on wetted items [150]. Use of black powder [118], chalk/silver powders combined with SDS [119], orange peels and rose petals [120] to dust fingermarks on items exposed to liquids [Note: in two of these studies [118,120], powder dusting was performed on porous substrates, which does not correspond to the recommended approach. The positive results were most likely due to the collection of sebum-rich fingermarks.].

**Water** – Four papers addressed the question of fingermark recovery on non-porous items [109,152,203] and porous items [113] submerged in water.

In the first study, Azman et al. compared the performance of a new powder suspension, based on the combination of a natural lipase and carbon nanotubes, with a commercially available SPR [152]. Depletion series of natural and sebum-rich (half-)fingermarks were left on three non-porous substrates (i.e., glass, plastic, aluminium) before being immersed in a pond for two and four weeks and subsequently processed. [Note: based on the methodological section, the way natural fingermarks were described and produced may indicate an improper interpretation of the existing guidelines.] Eventually, the authors concluded that both techniques performed equally.

In the second study, Abedi et al. aimed at assessing the performance of the sequence [CA fuming – powder dusting] to detect fingermarks on immersed items [203]. Depletion series of natural fingermarks were left by a single donor on different non-porous substrates (e.g. glass, stainless steel knife, laminated paper), which were subsequently immersed in a water-filled basin for one day up to 120 days. Fresh and seawater were considered, although no information was provided regarding the source of the seawater (i.e., natural or simulated). Once dried, the items were processed with the above techniques. [Note: a home-made fuming chamber without humidity control was used.] Overall, the authors concluded that the sequence [CA fuming – powder dusting] resulted in the recovery of fingermarks up to 90 days of immersion, with seawater being more detrimental compared to freshwater. The authors acknowledged that their conclusions may differ from most studies published on the topic, and that further experiments are required, taking into consideration parameters such as water flow and additional donors.

In the third study, Rivaldería et al. assessed the performance of the sequence [CA fuming – BY40] to detect fingermarks on immersed items [109]. Eccrine-rich fingermarks were deposited on three non-porous substrates (i.e., glass, plastic, aluminium) and aged for 24 h before being immersed in water-filled containers for one day up to 14 days. Fresh water and seawater were considered; both being collected from the natural environment. Once dried, the items were processed with the above sequence. Overall, the results showed that quality fingermarks could be recovered on all items up to 14 days of immersion. Aluminium yielded the best results and plastic the worst, the quality of the marks decreasing with the immersion time for all substrates. The authors also emphasized a difference of behaviour between water types, seawater resulting in higher quality fingermarks compared to fresh water.

Finally, Frick et al. investigated the impact of immersion on the recovery of fingermarks on porous substrates [113]. Sebum-rich (half-)fingermarks were left on copy paper and aged for 6 h before being immersed in water, considering four test configurations (i.e., fresh and salt water, both either still or moving). Fresh water was collected from the tap, and salt water was made by dissolving NaCl in tap water to reach a concentration of 3.5% (w/v). Moving water was simulated using pumps set to a 200 L/h flow rate. All the items were processed with either ORO or PD, while being wet. The results showed that ORO and PD were mostly unaffected by the immersion event, and that ORO outperformed PD regardless of the water type and immersion time. The authors noticed a colour shift of ORO (from red to orange) for items immersed for 20 days or more. They also emphasized a structural fragility of the paper immersed in moving salt water for longer times, resulting in item deterioration during the maleic acid step (PD). Finally, the authors acknowledged that some of their conclusions deviate from previous studies on the topic, which may be due to differences in methodological choices.

Overall, all these studies showed that fingermark recovery on immersed items is still possible by using conventional detection techniques. However, most of these studies considered sebum-rich fingermarks and controlled immersion conditions, resulting in almost all authors acknowledging the need for further studies on this topic.

**Corrosive substances** – Two studies addressed the impact of corrosive substances on fingermark recovery [204,205].

In the first study [204], depletion series of natural and sebum-rich fingermarks were deposited on glass slides and PET bottles before being immersed (immediately or after one week of aging) in a corrosive liquid (i.e., bleach, limescale remover, or lemon juice) for 1 min. Afterwards, the items were rinsed with water, air dried, and eventually processed with powder dusting (i.e., BMP or aluminium powder). Overall, all three substances resulted in major loss in fingermark detection capabilities, varying in intensity according to their nature (i.e., limescale being the most detrimental and lemon juice the least). The authors also emphasized differences of behaviour for the substrates, glass being more impacted by limescale remover and bleach, whereas PET was more impacted by lemon juice. They also observed differences induced by the fingermark composition, natural fingermarks being more impacted by the corrosive substances compared to sebum-rich ones. Finally, the detection performances of the control marks (i.e., not exposed to any corrosive substance) were reported, with 17% of good-quality natural marks (i.e., grade 3 or 4 on the CAST scale) and 81% of good-quality sebum-rich ones. This observation emphasized quite well the difference in detectability between natural secretions and sebum-rich ones.

In the second study [205], depletion series of natural (half-)fingermarks were deposited on three non-porous substrates (i.e., glass, PVC and HDPE) by a single *good* donor. Half-fingermarks were then aged for one day and two weeks before being immersed in a corrosive liquid (i.e., seven concentrated acids and three concentrated bases) for 1 min. Afterwards, the items were rinsed with water, air dried, and eventually processed with different detection techniques (i.e., BMP, Fe-BPS, Ti-WPS, CA fuming + BY40, VMD_Ag_). The corresponding halves were kept unexposed and acted as controls. Overall, all substances had a detrimental impact on the quality of the recovered fingermarks, with varying degrees according to their nature and concentration, concentrated sulfuric acid being the most damaging one. Regarding detection techniques, both PS were identified as the most effective techniques to process such items. On the other hand, CA fuming (+BY40) was determined to be the least effective. The authors also noted that (i) concentrated sulfuric acid was able to significantly damage PVC, (ii) glass resulted in the lowest detrimental impact, and (iii) items exposed to weaker acids may result in an increased ridge contrast when processed with Fe-BPS. Finally, the detection performances of the control marks were reported, with almost all techniques producing 50% of good-quality marks (i.e., grade 3 or 4 on the CAST scale), reflecting the good donorship character of the selected donor.

**CBRN decontaminants** – Radgen-Morvant et al. investigated the impact of CBRN decontaminants on fingermark recovery [206]. Natural (half-)fingermarks were left on glass slides and aged for two days before undergoing a decontamination process. Eleven decontaminations procedures were followed in this study: three aimed at removing the chemical agents through rinsing (water, soapy water, isopropanol), six aimed at neutralizing the chemical agents through chemical reactions (commercial liquids and gels), and two aimed at absorbing and neutralizing the chemical agents through particle coverage (commercial powders). The glass slides were then dried and observed using optical means. The results showed that the decontaminants in solid form had the least detrimental impact, followed with rinsing-based decontamination procedures. Liquid- and gel-based neutralizing decontaminants resulted in a massive loss of fingermark quality and detection capability (from 82% to 100% loss). The items were then processed with either CA fuming or MoS_2_-based SPR. Given that the results were strongly dependent of the decontaminant, the interested readers are encouraged to read the original paper, which provides detailed results for each one. Overall, both CA fuming and SPR resulted in an increase in fingermark quality and recovery, especially for fingermarks that were exposed to rinsing with (soapy) water or isopropanol. SPR outperformed CA for most decontaminants, especially for those relying on nucleophilic substitution. The only exceptions, for which CA outperformed SPR, were one of the powder-based decontaminants and one based on sodium hypochlorite. Despite those results, oxidative decontaminants were shown to be the most detrimental with a great number of fingermarks not recovered. [Note: it should be emphasized that this study is among the very few that considered the observation and recording of latent fingermarks through optical means before conducting the study.]

**Developed in other sections** – Performance of two ORO formulations on wetted papers [112] – See Section 3.3.5.

Acronyms used: **BMP** (black magnetic powder), **BY40** (Basic Yellow 40), **CA** (cyanoacrylate), **CAST** (Centre for Applied Science and Technology – UK), **CBRN** (chemical, biological, radiological, nuclear), **Fe-BPS** (iron oxide-based black powder suspension), **HDPE** (high-density polyethylene), **NP** (nanoparticle), **ORO** (Oil Red O), **PD** (physical developer), **PET** (polyethylene terephthalate), **PS** (powder suspension), **PVC** (polyvinyl chloride), **SDS** (sodium dodecyl sulphate), **SPR** (small particle reagent), **Ti-WPS** (titanium dioxide-based white powder suspension), **VMD** (vacuum metal deposition).

#### 3.5.3 Items exposed to heat

**Stainless steel and tinplate** – Zang et al. investigated the impact of heat on the ability to visualize latent fingermarks on stainless steel and tinplate, using coaxial illumination and CA fuming [177]. The study was based on natural fingermarks left on both metallic surfaces and exposed to temperatures ranging from 200°C to 800°C, for 10 to 30 min. On stainless steel, the authors showed that fingermarks started to become visible at 600°C and above, with minor impact of the heating time. The underlying metal also underwent colour change (from metallic to dark red, and eventually blue). Upon processing with CA fuming, the detection performances decreased as the heating temperature increased, with a major drop in fingermark quality observed at 600°C and above. On tinplate, the same trends were observed, but the quality of the detected fingermarks was poorer. Only an exposition to 800°C for 30 min led to clear fingermarks with coaxial illumination. Regarding CA fuming, fingermarks were detected up to 300°C, but a major drop in performance was observed at 400°C and above. Using optical microscopy and SEM observations, the authors explained their results by the evaporation and decomposition of organic compounds (such as amino acids) as the temperature increases, and a combination of oxidation and electrochemical corrosion of the steel surface at higher temperatures. Due to the tin layer present on the surface of tinplate, those chemical mechanisms were most likely hindered, explaining the lower detection performance on this substrate.

Acronyms used: **CA** (cyanoacrylate), **SEM** (scanning electron microscopy).

#### 3.5.4 Other contextual situations

**Frugal forensic science** – Bouzin et al. defined frugal forensic science [“frugal forensics”] as “the development of resilient and economical forensic science provision that meets the needs of society without compromising quality and safety” [207]. In their paper, the authors emphasized the need for research encompassing sustainability considerations and awareness about jurisdictions with limited resources or prone to supply chain disruption. They also proposed a grading system encompassing six parameters (i.e., performance, accessibility, availability, cost, safety, and simplicity) to determine if a detection method can be considered “sustainable”. This approach has been applied to amino acid reagents [90], powder dusting [134], or the processing of adhesives/tapes [146] – see Sections 3.3.1, 3.3.6, 3.4.2, respectively. In the same context, Lópes et al. reported the results of a survey conducted in Brazil to identify the challenges that fingermark detection practitioners face [208]. The key concern was the access to resources, equipment and routine materials, emphasizing the need for alternate and sustainable processes. The same applies to quality management systems, with standards hardly attainable or independent assessment unavailable [209].

**Post-blast IED** – Two papers addressed the question of fingermark recovery on detonated IEDs [58,210].

In the first study, two types of IEDs (i.e., RCIED and VOIED) and three explosives (i.e., TNT, C-4, AN-Al) were investigated [210]. Sebum-rich fingermarks were deposited by a single donor on the home-made IEDs and aged for five days before the devices were detonated. Both IEDs encompassed a plastic jug, a 9 V battery, and clear and black tapes. In addition to that, the VOIED included a metal can lid and wood, whereas the RCIED included a cell phone and a plastic switch. The post-blast materials were collected and processed with the following sequences: [preliminary examination – CA fuming – RAM] for non-porous items, [preliminary examination – NIN] for porous items, and [preliminary examination – CA fuming – PS (adhesive side) – RAM] for adhesives. Overall, 63% of the deposited latent fingermarks were recovered, mostly on VOIEDs and when using C-4. The highest recovery rates were obtained on metal and tapes (i.e., black vinyl and clear packing), and with CA fuming and PS. The authors acknowledged that such study would benefit from additional experiments, given that only one donor and sebum-rich fingermarks were used in this one.

In the second study, Kim et al. considered different scenarios involving the use of explosives (i.e., C-4, ANFO, PLX, TNT) to study the recovery of DNA and fingermarks on blasted items [58]. To reach that goal, an artificial secretion pad was used to deposit depletion series of fingermarks on a porous substrate (Kent paper) and a non-porous one (Ziplock bag). The fingermarks were then aged for an unspecified time before the IEDs were detonated. The post-blast materials were collected and processed according to conventional methods: IND/Zn for porous items, and [CA fuming – BY40] for non-porous items. The fact that the experiments were performed outdoor, without any containment area, resulted in several items not being recovered, mostly due to the wind. The authors successfully detected fingermarks on some of the recovered items, with higher quality fingermarks obtained on the non-porous substrates (compared to the porous ones).

**Exposure to dust** – Considering natural fingermarks left on mirrors, Accioly et al. showed that fingermarks exposed to airborne dust (e.g., coming from dry rural road) endure visual degradation which can prevent the observation of ridge details with optical means [196].

Acronyms used: **AN-Al** (ammonium nitrate combined with aluminium), **ANFO** (ammonium nitrate – fuel – oil), **BY40** (Basic Yellow 40), **CA** (cyanoacrylate), **DNA** (deoxyribonucleic acid), **IED** (improvised explosive device), **IND/Zn** (1,2-indanedione combined with zinc chloride), **MBD** (7-[p-methoxybenzylamino]-4-nitrobenz-2-oxa-1,3-diazole), **NIN** (ninhydrin), **PLX** (Picatinny Liquid Explosive), **PS** (powder suspension), **R6G** (Rhodamine 6G), **RAM** (mix of R6G, Ardrox P133D and MBD), **RCIED** (radio controlled IED), **TNT** (trinitrotoluene), **VOIED** (victim operated IED).

### 3.6 Quality assessment

#### 3.6.1 Quality control in laboratories

**Proficiency tests** – In the frame of an ENFSI-EU funded project, a study was conducted to provide forensic laboratories guidance for the selection of PTs in the fields of fingermark visualization, imaging, and comparison/identification [211]. To reach that goal, the authors purchased several PTs from different commercial providers, conducted them in their respective laboratories, and reported their observations and feedback in *ad-hoc* forms, which were eventually discussed to emit guidance. The paper includes an inventory of the PTs that were commercially available at the time of the project (i.e., 2022-2023). It also details the 30 guidance statements organized along three main categories: “Information available before purchasing the proficiency test” (11 statements), “Proficiency test setup and completion” (14 statements), and “Provider’s final report” (5 statements). The authors concluded that the overall status of the market was positive. Nevertheless, six major limitations (or lacks) were identified: incomplete information about the number of fingermarks and ten-print cards provided (COMP PTs), incomplete information about the resolution and format of the provided material (COMP PTs), unclear data management policy or compliance with the European GDPR (VIS and COMP PTs), difficulty level not reflecting the complexity of operational casework (VIS and COMP PTs), irrelevant tasks requested for VIS PTs, and conclusion scales not meeting the ENFSI guidelines (COMP PTs). The authors also emphasized that no PT could fully meet everyone’s expectations, for they are strongly related to the organization, the workload, and the expectations for such tests.

**Collaborative exercises** – Five papers reported the results of VIS CEs which took place in UK in 2020 [212] and in 2022-2023 [213], as well as in the frame of ENFSI-EU funded projects in 2019 [52], 2022 [214] and 2023 [214]. Generally, CEs are designed to provide a better understanding of the approaches followed by the participants when processing items (e.g., sequences, detection techniques, formulations, application protocols, observation methods). For this reason, most of the above-cited papers include a thorough analysis of the obtained results. Interested readers are redirected to the original papers for the detailed results and a complete overview of the observations made by the authors.

In the 2020 UK national CE, the participants were provided with a piece of illustrated wrapping paper, which can be characterized as a semi-porous substrate [212]. On each item, donors were asked to leave six fingermarks: four natural, one contaminated with butter (external greasy contamination), and one contaminated with copper traces through coins handling. This CE was designed to be difficult and to promote differences between participants. Overall, the authors identified numerous key learning points and needs. Only a few of those are cited here-after. First, the unequal awareness among participants regarding the flexibility of sequential processing and the need for training courses on the correct use of the UK Home Office FVM. Second, the need for improved notetaking to ease independent review or quality management. Finally, the requirement for some laboratories to update outdated processes. Indeed, 74% of the participants still applied DFO, despite the consensus about the superiority of IND/Zn. Also, among the participants who applied PD, 86% still used a formulation based on Synperonic N, a chemical banned in EU and no longer available for purchase. In terms of fingermark detectability, two fingermarks were detected by less than half the laboratories: a natural fingermark left on the non-porous side of the item and the copper-contaminated one. On the other hand, the butter-contaminated fingermark left on the non-porous side of the item and the natural fingermark left on the porous side were detected by all the participants, with varying degrees of ridge quality. Interestingly, the authors indicated that the same donor was at the source of both natural fingermarks cited above, which could indicate that the donorship level may be dependent of the detection technique.

In the 2022-2023 UK national CE, the participants were provided with a stainless-steel item bearing one fingermark left into blood (i.e., clean fingertip put in contact with blood present on the surface) and a piece of brown recycled paper bearing two fingermarks: one copper-contaminated one (see above) overlapped which a fingermark left by a bloody fingertip. The fingermark visualization step was combined with a comparison one, during which the participants were requested to provide comments at the activity level, more specifically regarding the deposition mechanism of the bloody fingermarks. The authors performed a very detailed interpretation of the results, pinpointing every deviation from the recommended sequences. They also identified numerous key learning points and needs. Only a few of those are cited here-after. First, DFO has been applied by 26% of the participants, compared to 73% for IND/Zn. Second, the processing of the porous item led to the largest deviation between participants, with 42% of them excluding the application of acid dyes after the amino acid reagents, whereas 58% applied at least one (i.e., AB or AV17). Finally, several participants used a pen to label visualized fingermarks directly on the items, which sometimes resulted in overlaps with ridge details detected afterwards. Alternative ways of marking fingermarks were advised by the authors.

In the frame of two ENFSI-EU funded projects, three multidisciplinary CEs were organized among the European community. The first one focused on DNA, fingerprints, documents and handwriting [52]. The participants were provided with a questioned envelope bearing two fingermarks (i.e., one inside and one covered with a stamp) and handwritten text, as well as with a letter bearing two fingermarks, handwritten text and indented impressions. The fingermarks were produced by using a stamp and a solution containing amino acids, with or without semen (for DNA). The second multidisciplinary CE covered the same fields as those described above [215]. The participants were provided with a questioned letter bearing handwritten text, a signature, a bloody fingermark, an eccrine-rich fingermark, a visible stain, and indented impressions. The third multidisciplinary CE focused on DNA, fingerprints, explosives, fibres and hair [214]. The participants were provided with a glass jar closed by a lid and eventually wrapped with black adhesive tapes. On the inside of the jar, the authors deposited traces of an explosive, a human hair and an eccrine-rich fingermark. On the outside, two sebum-rich fingermarks, animal hair and fibres were left on the adhesive side of the tapes, whereas saliva traces were deposited on both sides of the tapes. For the last two CEs, a particular attention was paid to promote points of contact between the different marks/traces, forcing the participants to setup a sequential recovery plan between the different fields of expertise. In all three papers, the numerous steps leading to the distribution of the CEs were thoroughly described (e.g., conceptualization, design, preparation, implementation and result evaluation). The authors also performed a very detailed description and interpretation of the results provided by the participants. If most of those were in line with the expected examination sequences, the authors emphasized some improvement key points. In the first CE, the authors emitted recommendations to improve the design and implementation of future multidisciplinary CEs. In the second CE, the authors underlined the sampling strategy (DNA and ink), when applied before fingermark detection, and the need for a higher consideration of indented impressions by participants. For the third CE, the authors discussed about the importance of preliminary observations to detect traces during the early stages of the sequence, the sampling strategy between fingermarks and explosive traces, the need to avoid the deposition of additional fingermarks by participants (mostly through gloves), and the need to reduce labelling discrepancies between the different fields.

**Positive controls** – Modified inkjet printers were used to produce positive controls (aka “test strips”) for different fingermark detection techniques [59]. A solution of 14 amino acids was printed on thick white paper to produce positive controls for amino acid reagents. An emulsion composed of four lipids (i.e., castor oil, stearic acid, palmitic acid, cholesterol), water, and Tween 20, was printed on thick white paper to produce positive controls for ORO and PD. Finally, a solution of sodium hydroxide was printed on acetate sheets to produce positive controls for CA fuming.

Acronyms used: **AB** (Amido Black), **AV17** (Acid Violet 17), **CA** (cyanoacrylate), **CE** (collaborative exercise), **COMP** (comparison), **DFO** (1,8-diazafluoren-9-one), **DNA** (deoxyribonucleic acid), **ENFSI** (European network of forensic science institutes), **EU** (European Union), **FVM** (fingermark visualization manual), **GDPR** (general data protection regulation), **IND/Zn** (1,2-indanedion combined with zinc chloride), **ORO** (Oil red O), **PD** (physical developer), **PT** (proficiency test), **VIS** (visualization).

#### 3.6.2 Fingermark quality assessment in research

**Foreword** – The question of fingermark quality assessment is crucial for any research taking place in the detection field. Currently, two main approaches coexist: the so-called “subjective” approach, relying on grading scales and human assessment, and the “objective” one, relying on metrics provided by quality assessment algorithms. The 2022-2025 period witnessed an increased interest for both approaches, with the publication of an extensive critical review about the use of grading scales, and several papers reporting the use of quality assessment algorithms in the detection field.

**Human-based grading** – Hanna et al. performed an extensive critical review about the use of grading scales by researchers between 1998 and 2022 [216]. They also conducted a survey among examiners and researchers to investigate the factors closely related to fingermark quality [217].

In their critical review, Hanna et al. covered several aspects related to the process of quality assessment in the detection field (e.g., historical and geographical considerations, degree of agreement with the IFRG guidelines, reflexions about the notion of “quality”) [216]. Given the size of their study, interested readers are redirected to the original paper for the detailed methodology and results, as well as for a complete overview of the observations made by the authors. In brief, the authors emphasized that the number of publications reporting the use of a grading scale has considerably increased during the last decade, with two commonly accepted absolute scales (i.e., CAST and UNIL) and a comparative one (i.e., UC). Among those three, the CAST scale was the most popular, with 40% of papers reporting its use. The authors also emphasized that “novel” scales, reported by 45% of papers, were mostly used in singular studies or by a single research group. Those tailored scales were mostly adapted from existing ones to introduce additional quality parameters or specific needs. With such a high number of different scales, it becomes difficult to compare studies or critically review emitted conclusions. Among their recommendations, the authors expressed the need for an agreement upon the definition of “quality”, to help decrease the need for tailored scales.

The survey conducted by Hanna et al. among fingerprint examiners and researchers aimed at giving an insight into the current parameters and factors deemed important to assess the quality of a fingermark [217]. The participants were asked to cite the parameters required to properly assess the effectiveness of a detection technique, to rank the importance of each parameter, to discuss the description of two common grading scales (i.e., CAST and UNIL) while being presented with images of fingermarks of varying quality, and to grade fingermarks by using continuous grading. Given the multiple scopes of this survey, interested readers are redirected to the original paper for the detailed methodology and results, as well as a complete overview of the observations made by the authors. In brief, the parameters with the highest rankings were ridge visibility, contrast, ridge detail, ridge flow and technique development. The CAST scale received the most responses of disagreement, especially for the description of the intermediate grading values. Participants also reported that the scale would benefit from including additional parameters such as ridge flow, contrast and ridge detail. On the other hand, the UNIL scale received the most responses in agreement, most likely because it considers more quality parameters than the CAST one. Participants reported ridge flow, ridge visibility and technique development as potential additional parameters. Finally, continuous grading introduced more subjectivity and scoring spread compared to categorical grading, with a strong impact of the participant’s training and experience. Among their recommendations, the authors expressed the need for a consistent categorical scale, to which up to two quality parameters could be added to fit the objectives of the research (e.g., “contrast” and “technique development” for pilot studies, “ridge detail” and “suitability for comparison” for casework implementation).

**Quality assessment algorithms** – The use of quality assessment algorithms commonly used in the comparison/identification field has been investigated by Bonnaz et al. as an alternative to the human grading process [97,218].

In their first study, Bonnaz et al. applied this approach to the comparison between two amino acid reagents (i.e., IND/Zn and DFO), for which a consensus exists in the literature [97]. Depletion series of natural (half-)fingermarks were left on five porous substrates by several donors (3′600 fingermarks in total) and aged for one day to one month before being processed. The fingermark images were then submitted to two quality assessment algorithms: LQM (Noblis/FBI) and ILFQM (IDEMIA). LQM provides 13 metrics, among which: overall quality and clarity of the fingermark, area of ridge flow of varying quality levels, and number of minutiae. ILFQM provides two metrics, among which: *expert_score*, which mimics the quality assessment of a fingermark if made by a fingerprint examiner. For both algorithms, significant differences in the distribution of scores obtained by IND/Zn and DFO were emphasized, in favour of the first one. This conclusion was in agreement with the consensus found in the literature.

In their second study, Bonnaz et al. investigated the impact of background illustrations on the fingermark quality assessment by automated algorithms [218]. To reach that goal, 480 items were collected (i.e., 240 porous and 240 non-porous, presenting – or not – illustrations) and distributed into eight background categories: S*ingle-color*, *Multi-color*, *Lines*, *Writings*, *Large letters, Rounds*, *Sinusoids*, and *Composite*. Natural fingermarks were left by various donors and aged for two days before being processed by one of the following sequential processes: [Preliminary observations – IND/Zn – NIN – PD] for porous substrates, [Preliminary observations – CA fuming – BY40/R6G/BMP] for non-porous substrates. The fingermark images were then submitted to two quality assessment algorithms: LQM (Noblis/FBI) and ILFQM (IDEMIA). The authors concluded that both algorithms performed best when the fingermarks were deposited on plain, uniform substrates. When illustrations are present, the algorithms could embed those as being part of the area of interest. In details, illustrations presenting limited contrast variations were assigned a low clarity grade, which resulted in a low or negligible impact on the provided scores. On the other hand, repetitive geometric illustrations or overlaps between highly contrasted area proved to be more problematic, with both algorithms tending to overestimate (LQM) or underestimate (ILFQM) the quality of the fingermarks. Overall, this study helped getting a better understanding of the performance and behaviour of both algorithms. It also helped refining the context into which such algorithms could be deployed in the detection field.

It should be noted that the use of quality assessment algorithms (more specifically: LQM) has also been used in some of the studies described in other sections of this report: evolution of ridge clarity along depletion series and aging processes [20,21,62], comparison between two formulations of a reagent [112]. Some studies also cited the use of such algorithms as a way to decrease the variability between assessors [89,110] or the workload [89].

Acronyms used: **BMP** (black magnetic powder), **BY40** (Basic Yellow 40), **CA** (cyanoacrylate), **CAST** (Centre for Applied Science and Technology – UK), **DFO** (1,8-diazafluoren-9-one), **IFRG** (International Fingerprint Research Group), **ILFQM** (improved latent fingerprint quality metric), **IND/Zn** (1,2-indanedione combined with zinc chloride), **LQM** (latent quality metric), **NIN** (ninhydrin), **PD** (physical developer), **R6G** (Rhodamine 6G), **UC** (University of Canberra – AU), **UNIL** (University of Lausanne – CH).

### 3.7 Impact of detection techniques on other forensic traces

**DNA** – A preliminary study proposed the use of non-thermal plasma as an alternative to UV-C for DNA decontamination of a VMD cabinet or other forensic devices [219]. Four studies aimed at assessing the impact of fingermark detection on DNA recovery, in terms of sequential processing [182,220,221] or decision-making [222].

In the first study, saliva stains were left on four non-porous substrates (i.e., plastic, metal, duct tape and rubber) and two porous ones (i.e., wood and paper) before being processed with various fingermark detection techniques [220]. Non-porous substrates were processed with the following sequence: [preliminary observations (incl. UV) – CA fuming – R6G – black powder]. Porous substrates were processed as follows: [preliminary observations – IND or DFO – NIN]. Overall, the authors concluded that the sequential process applied to the non-porous items did not affect DNA quantity and quality. On the other hand, NIN has significantly hampered DNA recovery on the porous items. The authors concluded that this reagent should be avoided if DNA is to be recovered.

In the second study, the impact of solvent-based adhesive removers on DNA recovery and amplification has been investigated [182]. To reach that goal, mock cases consisting in self-sticking stamps affixed on paper envelopes were considered. The items were processed as follows: [IND/Zn – NIN – Stamp removal – DNA recovery]. Stamp removal was conducted mechanically or by using an adhesive remover (i.e., Un-Du® or Turkish solution, which consists in mixing cyclohexane and isopropanol in a 1:2 v/v ratio) applied on the reverse side of the paper bearing the stamp. The authors showed that adhesive removal has no pronounced impact on DNA, provided that DNA has been purified or the volatile solvents evaporated before the amplification is carried out. Also, a transfer of material from the stamp to the enveloped has been observed, meaning that both surfaces should be sampled. Finally, significant loss of DNA was observed on the non-adhesive side of the stamp, which was supposedly caused by the application of amino acid reagents on a smooth surface.

In the third study, natural fingermarks were left by a single donor on black plastic bags, aged for an undetermined time before being processed by a single method (i.e., powder dusting) or by one of the following two sequences: [laser – RUVIS – CA fuming – RUVIS – RAM] or [CA fuming – RAM – laser – magnetic powder] [221]. The laser step consisted in irradiating the item for 10 s with a 532 nm wavelength (4 W). After DNA analysis, it was observed that powder dusting was extremely detrimental to DNA, with lower DNA yield, less alleles and lower peak heights as compared to unprocessed fingermarks. The same observation was made for the second sequential treatment. On the contrary, all the items processed with the first sequence, beginning with laser irradiation, led to more alleles and higher peak heights. More research is needed to explain this phenomenon.

Finally, Gardiner et al. aimed at gaining information about the way fingermark detection and DNA sampling should be sequentially applied [222]. Considering depletion series of sebum-rich fingermarks left by a single donor on plastic, the authors compared the following two sequences: [Fingermark deposition – DNA sampling – Powder dusting] and [Fingermark deposition – Powder dusting – DNA sampling]. Overall, the results showed that DNA sampling on unprocessed fingermarks has a substantial detrimental impact on fingermark recovery, DNA swab being more damageable than tape lift. Also, powder dusting had a detrimental impact on DNA recovery when compared to unprocessed fingermarks, white powder being more damageable than the black one. Nevertheless, in both cases, sufficient material (DNA or ridge details) remains for the subsequent processing step. The authors also noted no relationship between DNA quantification and fingermark quality.

**Polymer analysis** – The impact of fingermark detection techniques on the subsequent chemical analysis of polymeric materials has been investigated by Meikle et al. [197]. To reach that goal, PE resealable bags were processed with different combinations of CA fuming, dye staining (BY40 or R6G) and VMD (VMD_Ag/Zn_ and VMD_Ag/Zn/Au/Zn_). The bags were then analysed by FTIR spectroscopy and IRMS (carbon and hydrogen). With regards to FTIR, additional peaks originating from the CA fuming were observed, as expected. With regards to IRMS, the processed samples could not be discriminated from the unprocessed ones, meaning that the impact of the fingermark detection techniques was negligible for this technique.

Acronyms used: **BY40** (Basic Yellow 40), **CA** (cyanoacrylate), **DFO** (1,8-diazafluoren-9-one), **DNA** (deoxyribonucleic acid), **FTIR** (Fourier transform infrared), **IND** (1,2-indanedione), **IND/Zn** (1,2-indanedione combined with zinc chloride), **IRMS** (isotope ratio mass spectrometry), **MBD** (7-[p-methoxybenzylamino]-4-nitrobenz-2-oxa-1,3-diazole), **NIN** (ninhydrin), **PE** (polyethylene), **R6G** (Rhodamine 6G), **RAM** (mix of R6G, Ardrox P133D and MBD), **RUVIS** (reflected UV imaging system), **UV** (ultraviolet), **VMD** (vacuum metal deposition).

## Declaration of competing interest

The authors declare that they have no known competing financial interests or personal relationships that could have appeared to influence the work reported in this paper.
